# Conversion of Soluble Polyimines to Covalent Organic Framework Films and Composites

**DOI:** 10.1021/jacs.5c03079

**Published:** 2025-07-02

**Authors:** Ly D. Tran, Sachin Babu, Morgan E. Loveday, Vincent W. Chen, Dayanni D. Bhagwandin, John H. Dunlap, Kirt A. Page, Hilmar Koerner, Abigail T. Juhl, Christopher A. Crouse, Nicholas R. Glavin, Luke A. Baldwin

**Affiliations:** † Materials and Manufacturing Directorate, 33319 Air Force Research Laboratory, Wright-Patterson AFB, Ohio 45433, United States; ‡ BlueHalo, Dayton, Ohio 45431, United States; § Cornell High Energy Synchrotron Source, 5922 Cornell University, Ithaca, New York 14853, United States

## Abstract

Covalent organic frameworks (COFs) have been demonstrated for promising applications across research areas and industries. As research in the field advances, there is an increasing need for processing techniques for printing and fabricating COFs, as well as for synthesizing COF composites for advanced materials. To achieve this goal, a versatile approach allowing the synthesis of COFs through polyimines has been developed. Specifically, linear polyimines derived from an aliphatic diamine and aryl dialdehydes were synthesized and subjected to exchange reactions with various vertex amines to generate high-crystallinity imine-based COFs. These polyimines, also referred to as Schiff base polymers, are soluble in organic solvents, enabling solution-based processing and printing with vertex amines to create films of the desired shapes. Highly crystalline COF films were then fabricated by converting precursor films through vapor annealing. This process enables the fabrication of COFs on both microscopic and macroscopic scales. Furthermore, this method provides a straightforward approach to creating advanced functional materials, such as COF/CNT nanocomposites.

## Graphical abstract

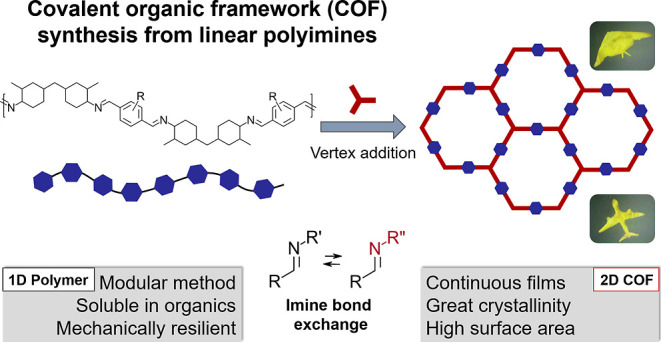

## Introduction

Covalent organic frameworks (COFs) are a class of crystalline, porous materials with high surface area and tunable pore size, shape, and functionality. These modular materials are composed of light-element organic nodes and edges connected by strong covalent bonds, which result in low-density materials exhibiting high stability in various environments. Since the first example reported in 2005 by Yaghi and co-workers, COFs have remained attractive materials for gas sorption, separation, catalysis, ionic conduction, energy storage, sensing, electronic, and optoelectronic devices.^1–3^ Within this research, there remains a crucial need to develop processing methods to synthesize COF films and perform complex fabrication tasks such as patterning and printing to further leverage the properties of these materials.

While COFs, as crystalline solids, present significant processing challenges due to their low solubility in solvents, there have been reports of printing and patterning COF colloidal inks, precursors, or monomers.^4–7^ Utilization of these advanced manufacturing techniques, however, requires that particle size and precipitation rate must be carefully controlled to limit the buildup of pressure and system clogging. When these features are appropriately controlled, colloidal COF inks have been demonstrated to be compatible with aerosol-jet printing^8^ or 3D flow-focusing microfluidic devices.^9^ There are also examples of custom-built inkjet printers that load monomer reagents into separate print heads to prevent rapid solidification of imine-based COFs.^10^ Recently, a photochemical approach integrated with a liquid flow system was used to synthesize patterned COFs through a dynamic liquid/solid interface.^11^ Although these prior studies represent significant advances, more general methods are needed to enable the fabrication of imine-based COF films by using solution-based techniques.

During imine-based COF synthesis, aldehyde and amine monomers quickly react to form amorphous polymers that then undergo error correction and structural reorganization to yield thermodynamically stable crystalline COFs.^12,13^ Leveraging this dynamic covalent chemistry, several studies have reported the conversion of small molecules, macromolecules, polymers, and membranes to COFs with improved crystallinity and surface area (as compared to their counterpart COFs prepared from aldehyde and amine starting materials).^14,15^ Specifically, aryl-protected imines, formed between terephthalaldehyde (PDA) or 2,5-dimethoxyterephthalaldehyde and substituted anilines, were used in place of aldehyde counterparts for the synthesis of COF-TP-Cl and COF-TD-Cl with shorter reaction times, higher yields, and larger surface area materials.^14^ Imine cages, constructed from the condensation of 1,3,5-triformylbenzene and 1,2-ethylenediamine, have also been converted to high-surface-area COFs at the aqueous–DCM interface under room temperature conditions.^15^ With regards to polymer-to-COF studies, a linear polyimine, obtained from *p*-phenylenediamine and PDA, underwent a linker replacement reaction with 2,4,6-triformylphloroglucinol to form β-ketoenamine COF-Tp, through irreversible tautomerization of an enol to the more stable ketone groups, which provides the driving force of the transformation.^16^ Linkage substitution can also be utilized to synthesize imide-based COFs from a polyimine since imide bonds are more stable and less reversible than imine bonds.^17^ While these reported works demonstrate the versatility of applying dynamic covalent bond exchange in COF synthesis, further investigation of the processability is warranted.

Linear polyimines, or Schiff base polymers, offer utility in functional polymer applications, and when incorporated into cross-linked networks, these materials possess the ability to be reprocessed, repaired, and recycled due to their reversible imine bonds. Polyimines are commonly synthesized through the condensation between amines and aldehydes, and the properties of the polymers can be tuned through careful choice of the monomers.^18–24^ In this work, polyimines derived from the polycondensation of rigid linkers and flexible aliphatic spacers create dynamic polymers that are soluble in organic solvents. In addition, due to the reversible nature of the imine bond, mixtures of the linear polyimines and vertex amines provide access to polymer films through common casting and printing techniques. Subsequently, the films can be converted to COFs through an annealing step. During this process, vertex amines undergo imine-amine dynamic bond exchange to replace aliphatic spacers and form defined shape, crystalline COF materials (Figure 1). Additionally, a composite material of COFs and carbon nanotubes (CNTs), COF/CNT, was prepared and showed significantly reduced resistivity, facilitating potential applications in electronic devices.

**Figure 1:**
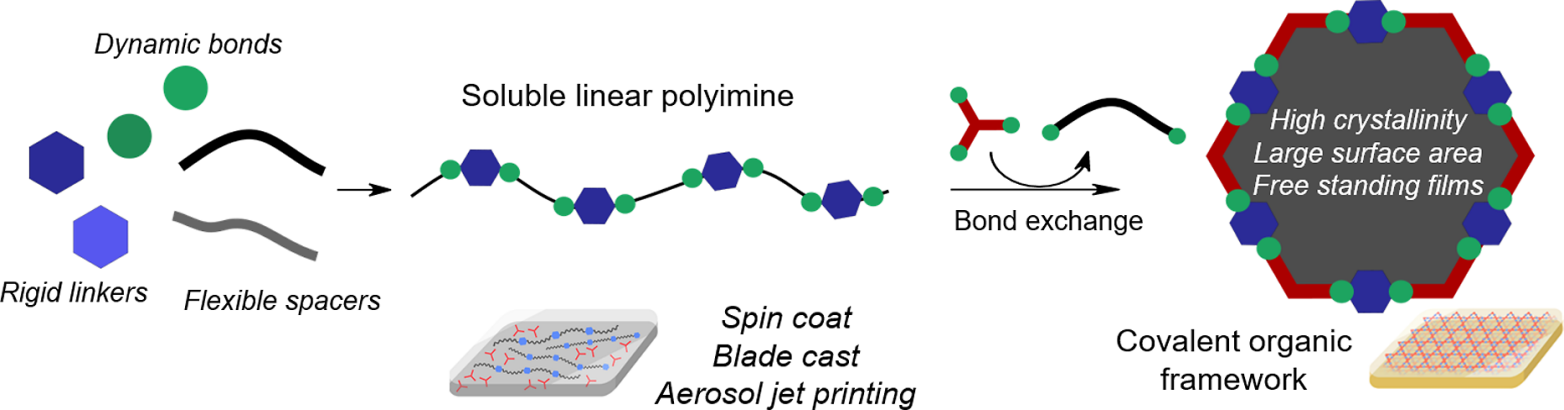
COF synthesis from polyimine through dynamic bond exchange.

## Results and Discussion

### Polyimine Synthesis

In this work, polyimines were synthesized through the condensation between 4MMCA (4,4′-methylenebis­[2-methylcyclohexanamine]) and PDA, or F4PDA, to create solution processable precursors for COF films (Figure 2). 4MMCA was selected as the aliphatic spacer, resulting in a polymer with both aliphatic and aromatic components to enhance solubility compared to all-aromatic linear polymers, which, while exhibiting successful dynamic bond exchange, suffered from insolubility and required conversion to more stable β-ketone linkages.^16^ The presence of the aliphatic component in the polymer and diamines ensured the synthesis of linear polymers with enhanced solubility to facilitate solution processing techniques. Initial experiments indicated that 4MMCA reacts with PDA to yield moderate *M*_w_ polyimine, as suggested by gel permeation chromatography (GPC) analysis (Figure S19). The polyimines 4MMCA-PDA and 4MMCA-F4PDA were formed under relatively mild conditions consisting of heating the monomers in THF at 64 °C for 4 h, followed by stirring overnight at RT (Figure 2a). After isolation, the presence of imine bonds in both polymers was confirmed by Fourier transform infrared (FTIR) spectroscopy with the presence of C=N stretching peaks at 1640 cm^–1^ and 1645 cm^–1^ for 4MMCA-PDA and 4MMCA-F4PDA, respectively (Figures S2 and S3). The depletions of C=O signals at 1683 cm^–1^ of PDA, 1699 cm^–1^ of F4PDA, and N–H stretching at 3362 cm^–1^ and 3288 cm^–1^ of free amine groups in 4MMCA indicate trace amounts of end groups in the polymer, suggesting a high degree of polymerization of the products. Similar to FTIR experiments, proton nuclear magnetic resonance (^1^H NMR) spectroscopy analysis of the polymers confirmed the presence of imine bonds in the product with the characteristic chemical shifts in the range of 8.4 to 8.15 ppm and 8.7 to 8.15 ppm for 4MMCA-PDA and 4MMCA-F4PDA, respectively (Figures S13 and S14). In both cases, the minimal presence of aldehyde end groups was validated by the ratios of aldehyde to imine peak which were calculated to be 1:46 and 1:71 for 4MMCA-PDA and 4MMCA-F4PDA, respectively (Figures S13 and S14), suggesting a higher degree of polymerization in the latter case (see the Supporting Information NMR section). In good agreement with NMR experiments, GPC analysis reveals the *M*_w_ values of 4MMCA-PDA and 4MMCA-F4PDA as 40.9 and 88.6 kDa, respectively, relative to polystyrene (Figures 2b and S19). Glass transition temperature (*T*_g_) of both materials were measured through differential scanning calorimetry (DSC) experiments and revealed *T*_g_ values of 190 °C for 4MMCA-PDA and 173 °C for 4MMCA-F4PDA (Figures 2b, S22, and S23). Thermogravimetric analysis (TGA) showed that under nitrogen, both polymers exhibited high thermal stability, with 4MMCA-PDA being stable up to 320 °C and 4MMCA-F4PDA being stable up to 330 °C (Figures S20 and S21). Unlike previous studies, where it is not possible to evaluate the mechanical properties of the polymeric COF precursors due to low solubility and processability, we were able to fabricate high-quality films for mechanical characterization. Particularly, both polymers exhibited good solubility in tetrahydrofuran (THF) and chloroform, making the polymers amenable to solution-based fabrication techniques. As a testament to this, slow evaporation of 4MMCA-PDA and 4MMCA-F4PDA solutions in THF were used to prepare uniform dog-bone-shaped films for mechanical testing (Figure 2b). Results from these tests indicate that 4MMCA-PDA and 4MMCA-F4PDA have elastic moduli of 1.27 ± 0.14 and 1.52 ± 0.33 GPa, ultimate tensile strengths of 72.0 ± 17.9 and 97.2 ± 16.4 MPa, and strains at break of 38.5 ± 14.4 and 42.8 ± 6.5%, respectively (Figures 3, S24, and S25). These values are comparable to those of reported aromatic linear polyimines.^20^ Collectively, the functionality and utility of the polyimine approach reported here are highlighted by the processing and mechanical tests that are made possible through the soluble linear polymer.

**Figure 2:**
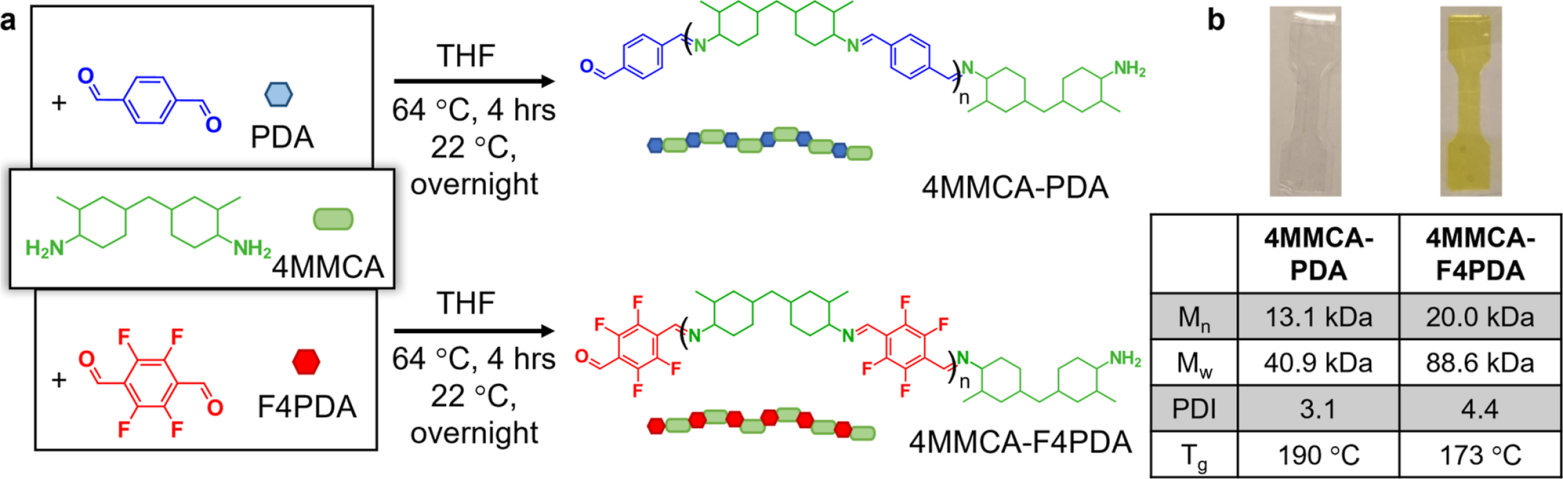
(a) Synthesis and characterization of newly reported polyimine in this work: 4MMCA-PDA and 4MMCA-F4PDA. (b) Summary of GPC analysis and thermal properties of 4MMCA-PDA and 4MMCA-F4PDA.

**Figure 3:**
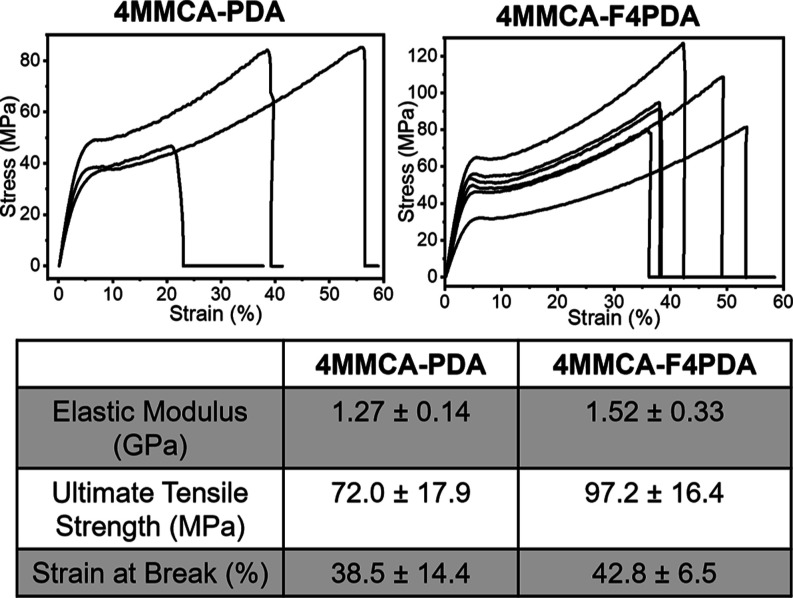
Mechanical property characterization of 4MMCA-PDA and 4MMCA-F4PDA.

### COF Powder Synthesis

As previously mentioned, an amine exchange reaction can be used to prepare crystalline COFs from small molecules, macromolecules, and polymers. However, to the best of our knowledge, this is the first time that 4MMCA polyimines have been reported and used in COF synthesis. Therefore, it was necessary to develop bond exchange conditions to explore the synthesis of COFs from these polymers. Within this exchange process, amine-derived components in the polyimine chain are replaced by new triamines, TAPB or TAPT, or tetraamine, ETTA. While incorporation of these rigid vertex units is required, the reaction mixture must also facilitate long-range ordering to form crystalline 2D sheets. In the absence of this, the materials would retain the new vertex but would be amorphous. Reaction optimization was performed for each target COF to generate materials with high crystallinity and porosity. TAPB-PDA is an established COF and has been demonstrated as a useful material in modern separation and filtration technologies.^25,26^ From polyimine 4MMCA-PDA, TAPB-PDA COF was synthesized through the reaction with TAPB using the commonly reported COF synthesis solvent system of 1,4-dioxane, mesitylene, and acetic acid at 70 °C for 72 h. While several COFs can be synthesized at room temperature, elevated temperatures enable high-crystallinity TAPB-PDA COF samples from polyimines (Figure S28). The obtained TAPB-PDA COF displayed excellent crystallinity as indicated by powder X-ray diffraction (PXRD), which showed Bragg peaks at 2.8° (100), 4.9° (110), and 5.6° (200).^12^ As expected, N_2_ sorption measurements at 77 K showed a type IV isotherm (Figure S33), indicating mesoporosity. Calculation from the isotherm reveals an extremely high surface area of 2032 m^2^/g (Figure 4b). This value is comparable with one of the best reported Brunauer–Emmett–Teller surface area (BET SA) measurements for TAPB-PDA COF and indicates that the polyimine-to-COF approach is a viable route to high-quality materials.^5,27^ Using similar synthesis conditions, TAPT-PDA COF was prepared from 4MMCA-PDA and TAPT. PXRD analysis for TAPT-PDA COF showed moderate crystallinity with its most intense Bragg peak at 2.9°, consistent with previously reported values.^28,29^ In contrast to TAPB-PDA, analysis from the N_2_ sorption isotherm gave a relatively low BET SA of 320 m^2^/g (Figure S34). It is worth mentioning that TAPT-PDA COFs are often reported with low surface areas (e.g., SA of 716 m^2^/g), potentially due to the imperfect stacking of 2D COF layers.^28,29^ Besides C_3_-symmetric linkers, TAPB and TAPT, which result in the formation of a hexagonal COF framework, this work also explored bond exchange of 4MMCA-PDA with C_2_-symmetric linker ETTA, which can yield either a rhombic or a Kagome structure framework. Unlike the cases of TAPB and TAPT, the reaction of 4MMCA-PDA with ETTA required heating at a higher temperature of 100 °C. The PXRD patterns of ETTA-PDA from this work matched with that of the Kagome structure framework with Bragg peaks at 2.7° (100), 5.4° (200), 8.1° (300), and 10.8° (400).^30^ Overall, ETTA-PDA also exhibited very good crystallinity and a high BET SA of 1822 m^2^/g (Figure S35), slightly higher than the value reported in traditional synthesis from the monomers of 1771 m^2^/g.^30^ Additionally, the fluorinated polyimine developed in this work, 4MMCA-F4PDA, was also reacted with TAPB through an exchange reaction to provide TAPB-F4PDA with good crystallinity and a moderate BET SA of 688 m^2^/g (Figures 4c and S36).^31^ Among these COFs, TAPB-F4PDA and TAPT-PDA exhibited suppressed BET surface area and their isotherms also contained hysteresis loops, which may be attributed to capillary condensation and/or pore blocking.^32,33^ FTIR analysis of these COFs showed the characteristic C=N stretching of imine bonds in the range of 1621 to 1626 cm^–1^, and the absence of C–H stretching peaks from 4MMCA at 2918–2921 and 2842–2869 cm^–1^ indicated that 4MMCA was completely exchanged with the new vertex amines (Figures S4–S7). Additionally, TAPB-PDA COF was hydrolyzed in DMSO–D_6_/DCl solution, and ^1^H NMR analysis of this solution indicated that there was no 4MMCA left after COF activation (Figures S15 and 16). These results highlight that imine-based COFs can be efficiently synthesized from the polyimines developed in this work. To further evaluate the new COF synthesis approach, monomer-derived COFs obtained from aldehydes and amines were synthesized and characterized by PXRD analysis. Results indicated that imine-based COFs synthesized from 4MMCA-derived polyimines have higher, or similar degrees, of crystallinity (Figures S29–S32). While previously reported procedures for synthesizing these COFs from aldehydes and amines required specific optimal conditions, these results suggest that the method developed here provides a more general approach across various COF structures.

**Figure 4:**
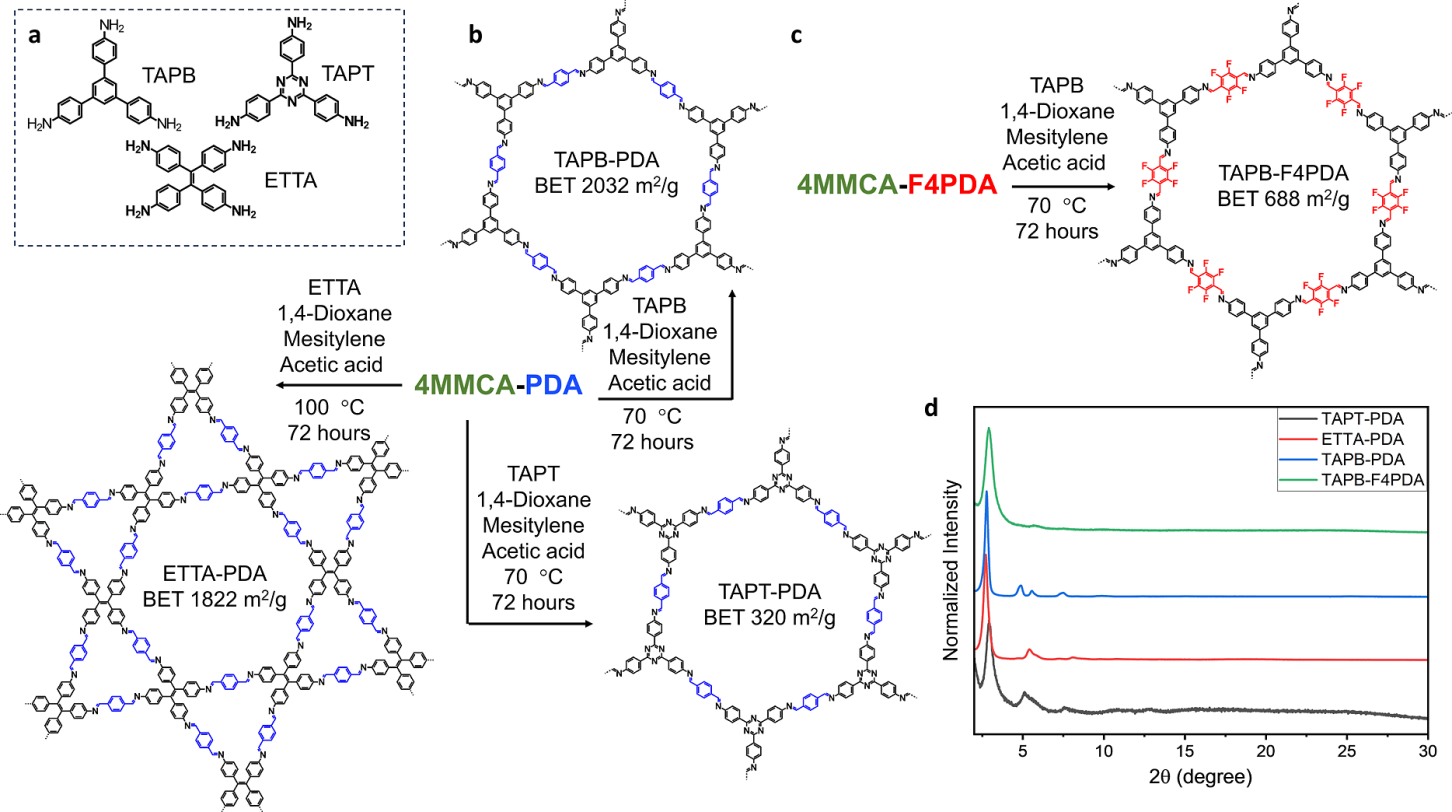
(a) Structures of vertex amines used in this study: TAPB, TAPT, and ETTA. (b) Synthesis and structures of TAPB-PDA COF, TAPT-PDA COF, and ETTA-PDA COF from 4MMCA-PDA. (c) Synthesis and structure of TAPB-F4PDA COF from 4MMCA-F4PDA. (d) Powder X-ray diffraction.

### COF Film Synthesis

Thin film fabrication is essential for practical applications of COFs in electronic and optoelectronic devices. Numerous approaches have been reported for COF film synthesis including bottom-up and top-down routes, interfacial synthesis, chemical vapor deposition, and interfacial-residual concomitance approaches.^6,34–37^ The 4MMCA-derived polyimines developed here are compatible with solution-based processing methods and are suitable precursors for the COF synthesis. Consequently, a two-step processing procedure to fabricate a COF film from the 4MMCA-PDA polymer was investigated. In the first step, 4MMCA-PDA and TAPB were dissolved in THF and cast onto a glass substrate by spin coating, drop casting, or blade coating methods. In the second step, the 4MMCA-PDA and TAPB film was converted to COF through heating in the vapor mixture of 1,4-dioxane, mesitylene, and acetic acid at 70 °C (Figure 5a). After 22 h of heating, a TAPB-PDA COF film with high crystallinity was obtained as indicated by grazing incidence wide-angle X-ray scattering (GIWAXS) analysis (Figure S26). The 1D projection of the GIWAXS pattern matches the PXRD pattern of the TAPB-PDA COF, validating the formation of the desired material (Figure S27). The GIWAXS image also indicated the random orientation of the film. Scanning electron microscopy (SEM) analysis was performed on films before and after conversion to a COF (Figure 5b). Results indicate a clear change in film morphology from a smooth polymer film to a rougher, plate-like COF film, validating the formation of crystalline COF materials after conversion. The relationship between time and early-stage film crystallinity was also explored using GIWAXS at a synchrotron source. Results indicate that crystallinity is quickly achieved in the first 30 min of the reaction^12^ and the crystallinity improves over time as indicated by increasing intensity of the GIWAXS signals (Figure 5c,d). After 3 h, other Bragg peaks with weaker diffraction intensity were recorded during the GIWAXS experiment, highlighting that this 2-step process generates highly crystalline COF films. The COF film after 3 h of conversion is relatively thin, attaches strongly to the substrate, and has a thickness of approximately 4 μm. To further explore the ability of fabricating a thick, stand-alone COF film, a modified procedure was developed. Specifically, the solution of 4MMCA-PDA and TAPB in THF was cast onto a glass substrate through a slow evaporation casting technique to form the precursor film, which was subsequently converted to TAPB-PDA COF film through exposure to solvents and heating (see “standalone thick COF film” part in the Supporting Information). Cross-sectional SEM analysis of this film showed a thickness of 114.5 ± 3.0 μm (Figure S47). Both PXRD and N_2_ sorption analyses confirm the crystallinity and porosity of the film (Figure S38). These experimental results demonstrate that through the polyimine precursor, COF films with various thicknesses ranging from microns to hundreds of microns can be prepared with good crystallinity and porosity.

**Figure 5:**
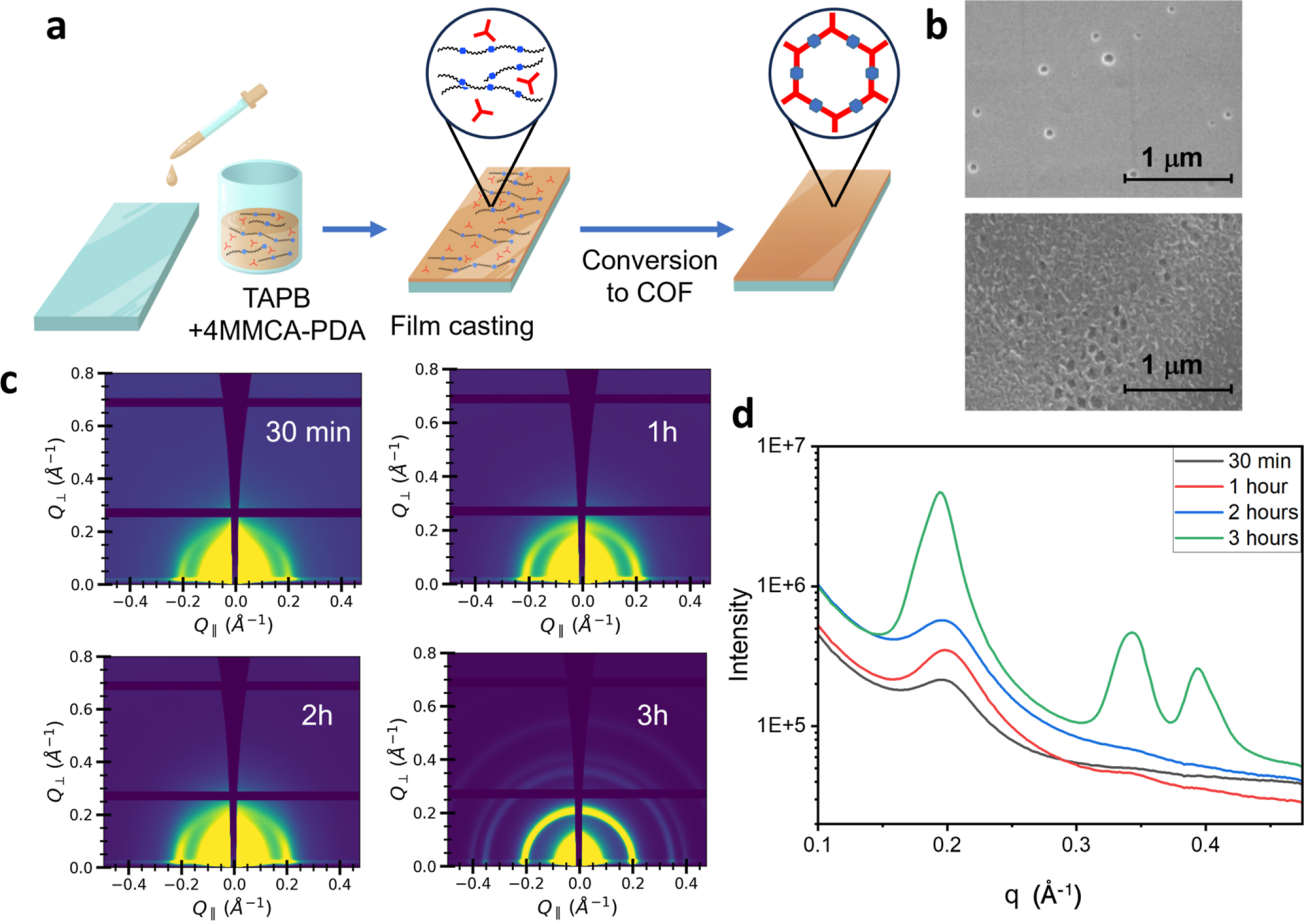
(a) Casting and synthesis of the TAPB-PDA film from TAPB and 4MMCA-PDA. (b) SEM images of the 4MMCA-PDA/TAPB film from blade cast (top) and TAPB-PDA COF film (bottom). (c) GIWAXS of the TAPB-PDA COF film from different reaction times. (d) 1D projection of GIWAXS scattering patterns.

### Functional Manufacturing

Fabricating COFs into the desired size and shapes is essential for applications in membrane filtration, separation, and electronic devices. However, this is often difficult to achieve due to the low processability of COFs. Conversion of 4MMCA-PDA polyimine to COFs represents an alternative method to exploit polymer processing and fabrication methods to increase shape complexity. An initial attempt to demonstrate this involved directly blade-coating a precursor mixture composed of TAPB and 4MMCA-PDA in THF onto a Teflon substrate. The resulting film from this procedure, however, was fragile and broke upon removal from the substrate. Since 4MMCA-PDA has good mechanical properties, a benefit of this linear polymer strategy, it was envisioned that this material could be used as a bottom layer to help mitigate film brittleness. In short, 4MMCA-PDA was cast on the Teflon substrate, followed by a top layer of a TAPB and 4MMCA-PDA mixture. As expected, this 2-layer film exhibited improved mechanical properties and facilitated large area film lift off from the substrate without significant breakage (Figure 6a) (up to 4 cm × 5 cm). The sample was subsequently laser-cut to obtain precursor films of the desired shapes (Figure 6a). As a demonstration to illustrate various shape complexities, the shape of a B2-Spirit aircraft, an airliner airplane, a circle, and a square were prepared. These films were then converted to COFs through heating in a vapor mixture of dioxane, mesitylene, and acetic acid at 70 °C overnight, similar to the film synthesis mentioned previously.^6^ After the reaction, TAPB-PDA COF was obtained, and PXRD characterization confirmed the crystallinity of these TAPB-PDA COFs (Figure 6d). SEM analysis of films before and after conversion reveals morphology changes from a smooth to a rough surface with plate-like structures (Figure 6b). It is worth mentioning that even though the COF conversion reaction involves the exchange of imine bonds in the 4MMCA-PDA polymer to imine bonds in TAPB-PDA COF, the shape of the object was retained after the reaction without noticeable breakage. To ensure that additional 4MMCA-PDA polymer was not left over from the 2-layer casting method, the TAPB-PDA COF film was hydrolyzed in DMSO–D_6_/DCl and analyzed by ^1^H NMR spectroscopy. The obtained spectra show no characteristic 4MMCA peaks in the aliphatic region of 3.0 to 0.5 ppm of the spectrum, which confirms that no appreciable 4MMCA remains in the material after washing (Figures S17 and S18). The result confirms that this method can be used to prepare imine-based COFs in desired shapes on a macroscopic scale (1 cm × 1 cm) with good precision. While the polyimines are resilient enough to get mechanical properties using traditional tensile testing, when converted to large area COF films, the materials exhibit brittleness as a result of the high crystallinity. This characteristic aligns with prior reports and highlights the challenges of creating thick, large-area films, along with the difficulty in testing. Frasconi and co-workers developed a novel, custom-made tensile testing platform to characterize the mechanical properties of large-area, free-standing TAPB-PDA COF specimens, demonstrating the strength and inherent brittleness of pure COF films. Their findings revealed that 85 nm COF nanofilms exhibited a high strength of 188 ± 57 MPa, a Young’s modulus of 37 ± 15 GPa, and a strain at break of 1.0 ± 0.3%.^38^

**Figure 6:**
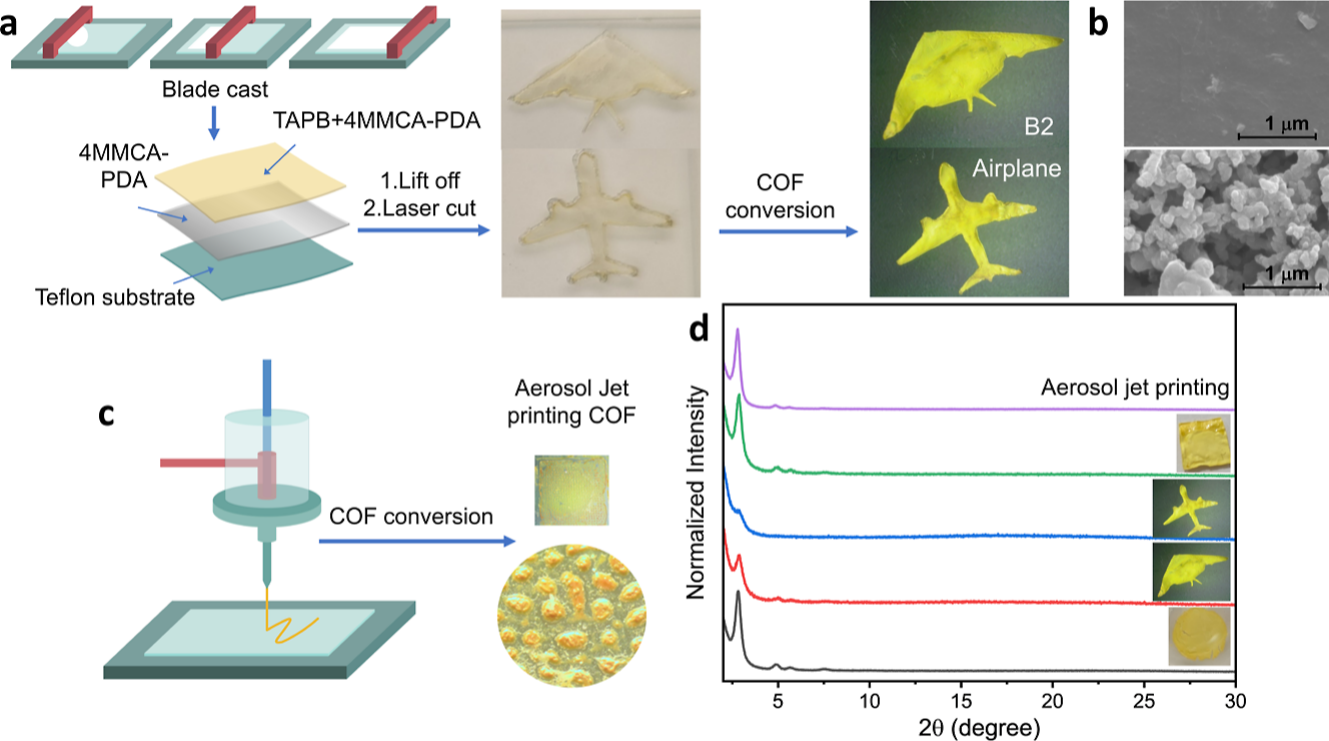
Process of fabricating the COF in desired shapes. (a) COF in B2 and airplane shape using blade-cast and laser-cut processing. (b) SEM images of top: the 2-layer precursor film (4MMCA-PDA bottom layer and 4MMCA-PDA/TAPB top layer) and bottom: TAPB-PDA COF film. (c) COF in a square shape fabricated through aerosol jet printing of the precursor mixture. (d) PXRD patterns of TAPB-PDA COF in various shapes obtained from film processing and aerosol jet printing.

To test the compatibility of polyimine-to-COF conversion with microscale processing, solution-phase aerosol jet printing was explored. As COFs tend to be very insoluble, there are only a few reported works with COF processing using aerosol jet printing.^8^ The polymer precursor mixture composed of TAPB and 4MMCA-PDA was dissolved in a chloroform/terpineol (9:1 by weight) solvent mixture and loaded into the ultrasonic atomizer of the printer (Figure 6c). Within this formulation, the aldehyde component of the linker is in a protected state, which helps to prevent rapid reactions with TAPB that would likely result in the formation of an insoluble TAPB-PDA polymer. This precursor solution was printed with a 300 μm nozzle at a flow rate of 35–50 sccm and a sheath gas flow rate of 50 sccm onto a 60 °C heated substrate into a 1 cm × 1 cm square. This was followed by heating the material in the vapor of 1,4-dioxane/mesitylene/acetic acid to convert the mixture to TAPB-PDA COF. After conversion conditions, formation of TAPB-PDA COF was confirmed by PXRD, which indicated a highly crystalline material (Figure 6d). While further optimization of printing conditions would be beneficial to control feature size, these results provide clear evidence that 4MMCA-derived polyimines enable a reliable method to harness solution processing to manufacture high-quality COFs.

### COF–CNT Composite

A large fraction of COFs exhibit low intrinsic electrical conductivity, limiting applications in electrochemistry and electronic devices. Incorporation of fillers to create composites with highly conductive nanomaterials can significantly improve the conductivity of the composites. Specifically, COF/CNT composites have demonstrated outstanding performance as energy storage materials,^39,40^ electrode materials for batteries,^41,42^ and materials for electrocatalytic reactions.^43,44^ In most of these prior reports, COF/CNT composites were synthesized through in situ growth of the COF on aminated or carboxylated CNTs. These methods used solvothermal reactions requiring lengthy synthesis procedures (i.e., 3 to 5 days).^40,42,44^ To test the compatibility of the polyimine-to-COF synthesis approach with composite film synthesis, a precursor mixture of 4MMCA-PDA and TAPB was combined with CNTs at different weight percentages (0.9%, 4.5%, and 8.7% CNT). Once combined in THF, sonication was used to help distribute the CNT within the 4MMCA-PDA/TAPB mixture. These solutions were then cast, and the solvent was allowed to slowly evaporate to generate polymer composite films of 4MMCA-PDA/TAPB/CNTs with defined CNT loadings. Conductivity measurements of these polymer/CNT films revealed a significant decrease in the resistivity of the films with increasing loading of CNTs. Particularly, 4MMCA-PDA/TAPB/CNTs with loadings of 0.9%, 4.5%, and 8.7% CNT exhibited resistivities of 2.3 × 10^4^, 97.2, and 2.0 Ω·cm, respectively. The CNT composite films were subjected to COF conversion conditions of heating in a vapor mixture of dioxane/mesitylene/acetic acid to convert the polymer composite to a TAPB-PDA COF/CNT composite. The resulting composites were denoted as COF/CNT-X where X indicates the initial loading of CNTs in the precursor films. The formation of crystalline TAPB-PDA COF was confirmed by PXRD analysis (Figure 7a). Cross-sectional SEM images of COF/CNT-0.9 and 4.5 composites were obtained, revealing the uniform distribution of CNTs within the materials (Figure 7b). Conductivity measurement of COF/CNT films gave the resistivity of 8.5 × 10^4^ and 15.2 Ω·cm for COF/CNT-0.9 and 4.5, respectively (Figure 7c). Attempts to synthesize a COF/CNT-8.7 composite resulted in the formation of a nonuniform film, highlighting the importance of having optimal CNT loading (Figures S42 and S50). Taking into consideration that the resistivity of nonconductive imine-based COF is at the level of 10^13^ Ω·cm, the COF/CNT-0.9 composite, derived from the precursor film with 0.9% loading of CNTs, decreased the resistivity of the material by 9 orders of magnitude.^45^ The fact that the resistivities of the precursor polymer/CNT films and the COF/CNT composite films are in the same order of magnitude with the similar loading of CNTs (0.9% and 4.5%) (Figure 7c) indicates that the quality of CNTs was not affected by the COF conversion reaction which is further supported by Raman spectroscopy analysis (Figures S39 and S40). This in turn provides a new route for the synthesis of functional composites for high-performance electronics applications.

**Figure 7:**
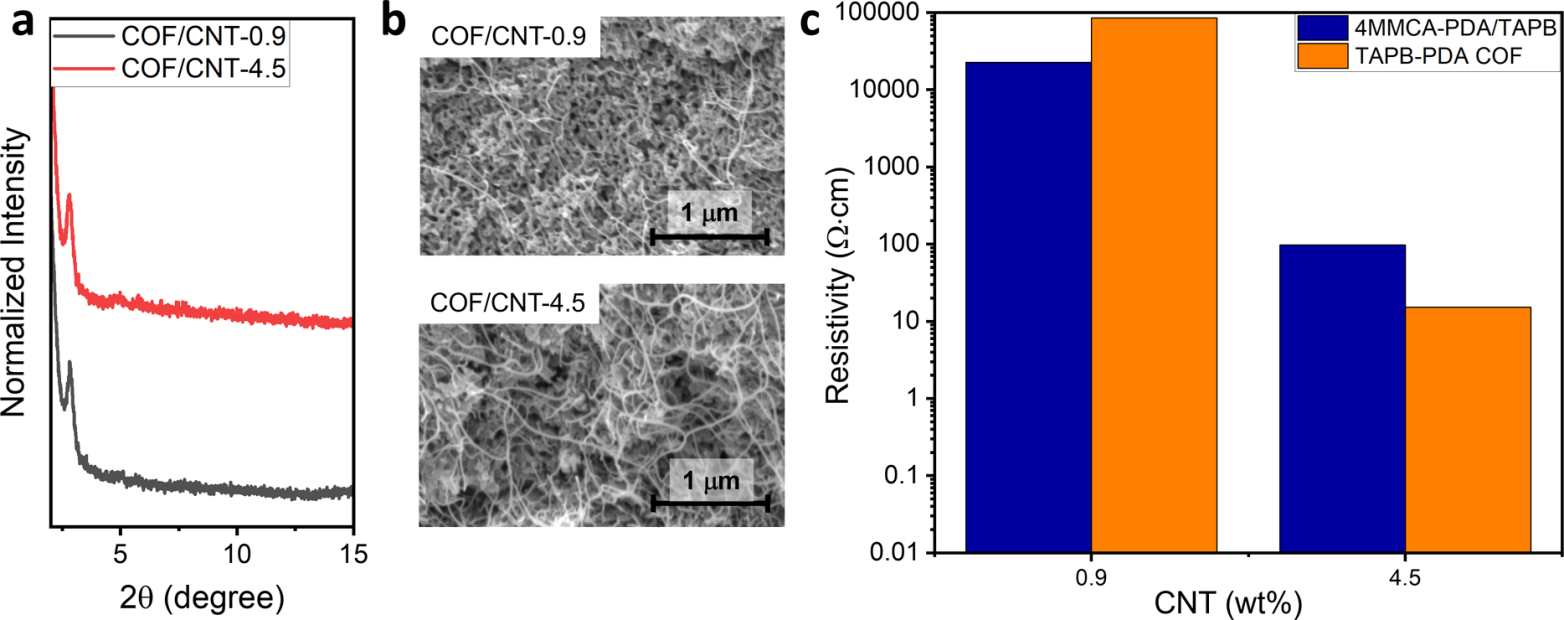
(a) PXRD patterns of TAPB-PDA COF composite films COF/CNT-0.9 and 4.5. (b) Cross-sectional SEM images of TAPB-PDA COF/CNT composites COF/CNT-0.9 (top) and 4.5 (bottom). (c) Resistivity of 4MMCA-PDA/TAPB/CNT films and TAPB-PDA COF/CNT composite films with 0.9 and 4.5 wt % of initial CNT loadings.

## Conclusions

In conclusion, the intrinsic nature of dynamic covalent chemistry within polyimines was leveraged to generate high-quality COFs. Specifically, polyimines 4MMCA-PDA and 4MMCA-F4PDA were shown to be excellent precursors to synthesize bulk COF powders or free-standing COF films. The reported polyimines offer excellent solubility in organic solvents, making common processing and printing techniques accessible for the fabrication of high-quality polymer-based films. Treatment of these samples with solvent vapor during heating, transformed films to highly crystalline imine-based COFs while also keeping the bulk shape intact. This method also showed great tolerance to the incorporation of a nanofiller for the creation of functional composites. As one such example, a COF/CNT-4.5 composite synthesized in this work exhibited a resistivity of 15.2 Ω·cm, 12 orders of magnitude lower than the resistivity of a nonconductive imine-based COF.

## Supplementary Material



## Notes

Experimental procedures, spectral data, and further characterization of compounds (PDF)
