# Supplementary material for: Conversion of Soluble Polyimines to Covalent Organic Framework Films and Composites
Source: J Am Chem Soc. 2025 Jul 2;147(28):24413–21. doi: 10.1021/jacs.5c03079 (PMC12980828; doi:10.1021/jacs.5c03079)
Supplement: Supplementary file 1 [file ja5c03079_si_001.pdf]

Supporting Information

Conversion of Soluble Polyimines to Covalent Organic Framework Films and Composites

Ly D. Tran,*[,a] Sachin Babu,[a,b] Morgan E. Loveday,[a,b] Vincent W. Chen,[a,b] Dayanni D. Bhagwandin,[a,b] John H. Dunlap,[a,b] Kirt A. Page,[a,b,c] Hilmar Koerner,[a] Abigail T. Juhl,[a] Christopher A. Crouse,[a] Nicholas R. Glavin,[a] Luke A. Baldwin*[,a]

[a]Materials and Manufacturing Directorate,
Air Force Research Laboratory, Wright-Patterson AFB, OH 45433, USA
[b]BlueHalo, Dayton, OH 45431, USA
[c]Cornell High Energy Synchrotron Source,
Cornell University, Ithaca, NY 14853, USA

*Email: Luke.baldwin.1@us.af.mil; ly.tran.5@us.af.mil

Table of Contents

General Considerations 2
Material Synthesis 4
Fourier Transform Infrared Spectroscopy 11
Nuclear Magnetic Resonance Spectroscopy 14
Gel Permeation Chromatography (GPC) 22
Thermogravimetric Analysis (TGA) 23
Differential Scanning Calorimetry (DSC) 24
Stress-Strain Analysis 25
X-ray Diffraction 27
Gas Sorption 31
Raman Spectroscopy 36
Resistivity Measurement 38
Scanning Electron Microscopy 39
References 42

General Considerations: All reagents were obtained from commercial vendors and used as-received unless otherwise noted.

FTIR IR Spectroscopy was performed using a Bruker Invenio X in ATR mode using solid powders or liquid drops of materials.

NMR ^{1}H-NMR spectra were obtained on Bruker 400 spectrometer using TMS as a standard.

PXRD: Powder X-ray diffraction data were collected on a Rigaku Smartlab diffractometer. The Cu-Kα(1.5406 Å) X-ray radiation source was operated at 40 kV and 44 mA. Samples were evenly dispersed on a standard silicon wafer from Rigaku. Samples were then transferred to the diffractometer and analyzed under ambient conditions.

Grazing-Incidence Wide-Angle X-Ray Scattering (GIWAXS)[1,2] performed at CHESS: The grazing-incidence X-ray scattering measurements were carried out at the Functional Materials Beamline (FMB) of the Materials Solutions Network at the Cornell High Energy Synchrotron Source (MSN-C). An X-ray beam energy of 9.7 keV (l=1.28 Å) was selected using the 111 reflection of a single-bounce, HPHT diamond monochromator. Harmonic rejection and vertical focusing are provided by a 1-meter long, bendable, rhodium-coated monochromatic mirror located approximately 7 meters upstream of the experimental hutch at an incident angle of 4 milliradians. Experiments were carried out in "bulk-beam" mode and the monochromatic mirror was used to focus the beam into a spot approximately (0.045 x 0.5) mm^2 at the sample position, with a total flux of approximately 10^{12} photons/second at 125 mA beam current. The samples were mounted on a 4-axis goniometer and aligned using a downstream ion chamber. Experiments were performed over a range of incident angles, both below and above the film critical angle. Scattering images were collected on a Pilatus 300K detector (Dectris, Baden, Switzerland) with a sample-to-detector distance of ca. 42.3 cm. Detector images were calibrated using silver behenate to convert the images to q-space. Python code was used to correct and analyze the scattering images and to produce intensity versus scattering vector, $Q_{||}$(Å-1), plots.

GIWAXS performed at AFRL: Grazing-incidence GIWAXS measurements were performed with a Xeuss 3.0 (XENOCS, France) system. The system is equipped with a Genix 3D (The Cu-Kα) X-ray radiation source and a Pilatus3R 300K detector (Dectris, Switzerland). The sample-to-detector distance was set at 70 mm and the grazing incident angle was set at 0.15˚. Experiments were performed under vacuum atmosphere.

CNT: Signis Single-wall Carbon Nanotubes (SWCNTs) were purchased from CHASM (CG100, Economical Single-Wall Carbon Nanotubes).

Raman Spectroscopy: Raman spectroscopy was performed using a Renishaw inVia Raman microscope under 785 nm excitation (to minimize fluorescence of the COF/polymer), with 1% laser power and 1 second exposure time. The incident laser beam was focused using a 50x long range objective lens to prevent contact of the lens with sample films. Films were positioned on top of double-side polished silicon wafers during measurement

Gas sorption measurements: N_2 sorption experiments were carried out using an ASAP 2020 (Micromeritics, Norcross, Georgia, USA). He (99.999%, used to determine void volume), Argon (99.999% purity) were purchased from Weiler Welding Co. Inc., N_2 (99.999% purity) was purchased from Indiana Oxygen Company and used as received. An activated sample (70-120 mg) was charged into a sample cell subsequently transferred to the degas unit for degassing and sorption apparatus for measurement at 77 K (N_2 isotherm), 273 K (CO_2 isotherm).

Elemental analysis was performed at Galbraith Laboratories, TN.

Profilometer: Film thickness was measured by DektakXT® stylus profilometer (Bruker, Billerica, MA, USA)

TGA: A thermogravimetric analyzer was used to measure the degradation temperature of the polyimines. A TGA5500 (TA Instruments, Delaware USA) was used to perform the analyses. A platinum (Pt) 100µL pan was tared by the instrument, after which a small amount of sample material (< 10 mg) was placed in the pan, weighed and placed in the furnace. The sample material was heated at a rate of 10 °C·min^{-1} up to 1000 °C in nitrogen.

DSC: Differential scanning calorimetry (DSC) was used to measure the glass transition temperature, T_g, of the polyimines. A DSC2500 (TA Instruments, Delaware USA) was used to perform the analyses. Tzero Aluminum (Al) hermetically sealed pans were used for both reference (empty) and sample. A standard Heat Cool Heat experiment was run in nitrogen for both polyimines. The following steps were used:

1. Equilibrate the pans to -50 °C.
2. Heat both the reference and sample pans at a rate of 10 °C·min^{-1} up to 250 °C.
3. Cool both the reference and sample pans at a rate of 10 °C·min^{-1} up to 250 °C.
4. Heat both the reference and sample pans at a rate of 10 °C·min^{-1} up to 250 °C.

DMA: To perform stress-strain analysis on the two polyamine materials, the ASTM D638 Type V standard dogbone was used. As permitted by the standard, the Type V template was scaled to accommodate the tensile clamp dimensions. The dogbones were laser cut (Epilog Fusion Pro Model 17000) from the polyimine thin films. The gage length, width, and thickness were around 9.5 mm, 2.6 mm, and 0.04 mm, respectively.

A dynamic mechanical analyzer, DMA800 (TA Instruments, Delaware USA), in tensile mode using a single screw film/fiber clamp, was used to perform the stress-strain tests. The samples were mounted to the clamps with a preload force of 1mN. The fixed sample was conditioned to the temperature of 20 C with a soak time of 1 min, after which the sample was extended at the displacement rate of 1.0 mm·min^{-1} up to 10 mm. The sample would break well before than 10 mm limit.

Scanning Electron Microscopy (SEM): SEM image collection was conducted using a Thermo Fisher Scientific Apreo C FEG scanning electron microscope, operated at 5 kV with probe current of 0.10 nA. Prior to SEM analysis the samples were sputter coated with 5 nm of AuPd to provide electrical conductivity.

Material Synthesis

Polymer synthesis

4MMCA-PDA

To a 40ml vial was added 4MMCA (Bis(4-amino-3-methylcyclohexyl)methane) (152 mg, 0.64 mmol, 1 equivalent) and 8 ml of anhydrous THF. To a 20 ml vial was added terephthalaldehyde (PDA) (86 mg, 0.64 mmol, 1 equivalent) and 8 ml of anhydrous THF. Both 4MMCA and PDA solution were sonicated (~ 30 seconds) to dissolve both compounds. PDA solution was transferred to 4MMCA solution with stirring and the vial was rinsed with 4 ml of THF. The reaction was heated to 64 °C for 4 hours with stirring and kept at room temperature for 22 hours (overnight). After completion, the solvent was removed by rotavap to almost dryness (~0.5 ml THF left). The 4MMCA-PDA polyimine product was crashed out in MeOH (~20 ml) and left in the fridge for a few hours followed by filtration and washing by MeOH. The dry product was transferred to a pre-weight vial and dry under house vacuum overnight to give 195.9 mg of white product (91% yield). FT-IR (cm $^{-1}$) 2918, 2842, 1640, 1446, 1371, 1295, 959, 848, 828, 475. Elemental analysis $C_{23}H_{32}N_2$ calculated value C 82.09, H 9.59, N 8.32; Found C 81.38, H 9.39, N 8.16.

4MMCA-F4PDA

To a 40 ml vial was added 4MMCA (Bis(4-amino-3-methylcyclohexyl)methane) (477 mg, 2.0 mmol, 1 equivalent) and 8 ml of anhydrous THF. To a 20 ml vial was added tetrafluoro terephthalaldehyde (F4PDA) (412 mg, 2.0 mmol, 1 equivalent) and 8 ml of anhydrous THF. Both 4MMCA and F4PDA solution were sonicated (~ 30 seconds) to dissolve both compounds. F4PDA solution was transferred to 4MMCA solution with stirring and the vial was rinsed with 4 ml of THF. The reaction was heated to 64 °C for 4 hours with stirring and kept at room temperature for 22 hours (overnight). After completion, the solvent was removed by rotavap to almost dryness (~0.5 ml THF left). The 4MMCA-F4PDA polyimine product was crashed out in MeOH (~100 ml) and left in the fridge for a few hours followed by filtration and washing by MeOH. The dry product was transferred to a pre-weight vial and dry under house vacuum overnight to give 760.1 mg of white product (93% yield). FT-IR (cm $^{-1}$) 2921, 2869, 1645, 1635, 1479, 1461, 1456, 1390, 1387, 1373, 1353, 1302, 1016, 997, 975, 959, 917, 667, 658. Elemental analysis $C_{23}H_{28}F_4N_2$ calculated value C 67.63, H 6.91, N 6.86; Found C 67.52, H 6.65, N 6.80.

Scheme S1. Reaction of amine containing siloxane and PDA that results in low molecular weight product

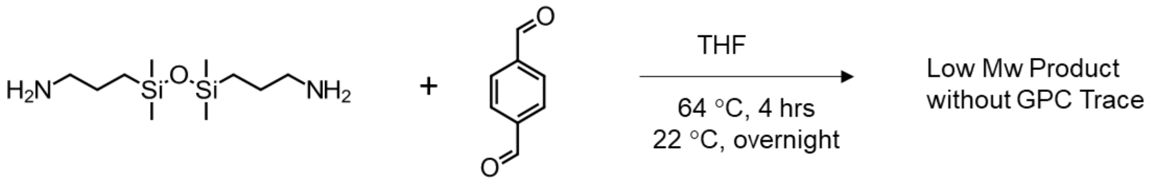

COF powder synthesis

TAPB-PDA COF from 4MMCA-PDA and TAPB

To a 7 ml vial was added 4MMCA-PDA (43 mg, 0.13 mmol, 1.5 equivalent), TAPB (30 mg, 0.085 mmol, 1 equivalent), and 1,4-dioxane/mesitylene solution (4:1 v/v, 1.5 ml). Resulting mixture was sonicated for 15 mins to dissolve all components followed by heating to 70 °C for 10 mins. Acetic acid 10.5 M (0.69 ml) was then added and the reaction was heated to 70 °C for 3 days. After completion, the reaction was cooled down to RT and the product was washed with MeOH and acetone. The product was stored in acetone until activation.

Activation: COF in acetone was exchanged to DCM and n-hexane. n-Hexane was decanted out of the vial and the vial was then placed under the constant flow of N_2 and was heated to 150 °C for 1.5 hours and kept at 150 °C for 3 hours, followed with cooling down to RT for 30 mins and placed under house vacuum overnight. This process yielded an activated TAPB-PDA COF as a yellow color solid (39.8 mg).

TAPT-PDA COF from 4MMCA-PDA and TAPT

To a 20 ml vial was added 4MMCA-PDA (202 mg, 0.60 mmol, 1.5 equivalent), TAPT (142 mg, 0.40 mmol, 1 equivalent), and 1,4-dioxane/mesitylene solution (4:1 v/v, 7 ml). Resulting mixture was sonicated for 15 mins followed by heating to 70 °C for 10 mins with stirring. Acetic acid 10.5 M (3.3 ml) was then added and the reaction was heated to 70 °C for 3 days. After completion, the reaction was cooled down to RT and the product was washed with MeOH and acetone. The product was stored in acetone until activation.

Activation: Same procedure as TAPB-PDA COF powder. This process yielded an activated TAPT-PDA COF as a yellow color solid (114.6 mg).

ETTA-PDA from 4MMCA-PDA and ETTA

To a 15 ml pressure vessel was added 4MMCA-PDA (100 mg, 0.30 mmol, 2 equivalent), ETTA (60 mg, 0.15 mmol, 1 equivalent), 1,4-dioxane (1.6 ml), and mesitylene (0.4 ml) . Resulting mixture was sonicated for 30 mins followed by heating to 100 °C for 10 mins with stirring. Acetic acid 6M (0.5 ml) was then added and the reaction was heated to 100 °C for 3 days. After completion, the reaction was cooled down to RT and the product was washed with MeOH and acetone. The product was stored in acetone until activation.

Activation: Same procedure as TAPB-PDA COF powder. This process yielded an activated ETTA-PDA COF as a yellow color solid (60.6 mg).

TAPB-F4PDA from 4MMCA-F4PDA and TAPB

This synthesis used 2 of 7ml vials. To each of the vial was added 4MMCA-F4PDA (53 mg, 0.129 mmol, 1.5 equivalent), TAPB (30 mg, 0.085 mmol, 1 equivalent), and 1,4-dioxane/mesitylene solution (4:1 v/v, 1.5 ml). Resulting mixture was sonicated for 15 mins followed by heating to 70 °C for 10 mins with stirring. Acetic acid 10.5 M (0.69 ml) was then added and the reaction was heated to 70 °C for 3 days. After completion, the reaction was cooled down to RT and the product was washed with MeOH and acetone. The product was stored in acetone until activation.

Activation: COF in acetone was exchanged to DCM and n-hexane. Products from 2 vials were combined. n-Hexane was decanted out of the vial, and the vial was then placed under the constant flow of N_2 and was heated to 150 °C for 1.5 hours and kept at 150 °C for 3 hours, followed with cooling down to RT for 30

mins and placed in house vacuum overnight. This process yielded an activated TAPB-F4PDA COF as an orange color solid (90.5 mg).

TAPB-PDA COF from 4MMCA-PDA and TAPB at room temperature (RT)

To a 4 ml vial was added 4MMCA-PDA (15 mg, 0.044 mmol, 1.5 equivalent), TAPB (10 mg, 0.0297 mmol, 1 equivalent), and 1,4-dioxane/mesitylene solution (4:1 v/v, 0.5 ml). Resulting mixture was sonicated for 15 mins to dissolve all components followed by resting at RT for 10 mins. Acetic acid 10.5 M (0.23 ml) was then added and the reaction was left at RT for 3 days. After completion, the product was washed with MeOH and acetone. The product was stored in acetone until activation.

Activation: COF in acetone was exchanged to DCM and n-hexane. n-Hexane was decanted out of the vial and the vial was then placed under the constant flow of N_2 and was heated to 150 °C for 1.5 hours and kept at 150 °C for 3 hours, followed with cooling down to RT for 30 mins and placed under house vacuum overnight. This process yielded an activated TAPB-PDA COF as a yellow color solid (15.1 mg).

TAPB-PDA COF from PDA and TAPB

To a 20 ml vial was added PDA (57 mg, 0.43 mmol, 1.5 equivalent), TAPB (100 mg, 0.284 mmol, 1 equivalent), and 1,4-dioxane/mesitylene solution (4:1 v/v, 5 ml). Resulting mixture was sonicated for 15 mins to dissolve all components followed by heating to 70 °C for 15 mins with stirring. Acetic acid 10.5 M (2.3 ml) was then added and the reaction was heated to 70 °C for 3 days. After completion, the reaction was cooled down to RT and the product was washed with MeOH and acetone. The product was stored in acetone until activation.

Activation: COF in acetone was exchanged to DCM and n-hexane. n-Hexane was decanted out of the vial and the vial was then placed under the constant flow of N_2 and was heated to 150 °C for 1.5 hours and kept at 150 °C for 3 hours, followed with cooling down to RT for 30 mins and placed under house vacuum overnight. This process yielded an activated TAPB-PDA COF as a yellow color solid (131.8 mg).

TAPT-PDA COF from PDA and TAPT

To a 20 ml vial was added PDA (57 mg, 0.43 mmol, 1.5 equivalent), TAPT (101 mg, 0.284 mmol, 1 equivalent), and 1,4-dioxane/mesitylene solution (4:1 v/v, 5 ml). Resulting mixture was sonicated for 15 mins followed by heating to 70 °C for 15 mins with stirring. Acetic acid 10.5 M (2.3 ml) was then added and the reaction was heated to 70 °C for 3 days. After completion, the reaction was cooled down to RT and the product was washed with MeOH and acetone. The product was stored in acetone until activation.

Activation: Same procedure as TAPB-PDA COF powder. This process yielded an activated TAPT-PDA COF as a yellow color solid (128.4 mg).

ETTA-PDA COF from PDA and ETTA

To a 15 ml pressure vessel was added PDA (82 mg, 0.61 mmol, 2 equivalent), ETTA (120 mg, 0.3 mmol, 1 equivalent), 1,4-dioxane (3.2 ml), and mesitylene (0.8 ml) . Resulting mixture was sonicated for 15 mins followed by heating to 100 °C for 15 mins with stirring. Acetic acid 6M (1 ml) was then added and the reaction was heated to 100 °C for 3 days. After completion, the reaction was cooled down to RT and the product was washed with MeOH and acetone. The product was stored in acetone until activation.

Activation: Same procedure as TAPB-PDA COF powder. This process yielded an activated ETTA-PDA COF as a yellow color solid (105.9 mg).

TAPB-F4PDA from F4PDA and TAPB

To a 20 ml vial was added F4PDA (88 mg, 0.43 mmol, 1.5 equivalent), TAPB (100 mg, 0.284 mmol, 1 equivalent), and 1,4-dioxane/mesitylene solution (4:1 v/v, 5 ml). Resulting mixture was sonicated for 15 mins followed by heating to 70 °C for 15 mins with stirring. After sonication, large precipitate was formed. Acetic acid 10.5 M (2.3 ml) was then added and the reaction was heated to 70 °C for 3 days. After completion, the reaction was cooled down to RT and the product was washed with MeOH and acetone. The product was stored in acetone until activation.

Activation: Same procedure as TAPB-PDA COF powder. This process yielded an activated TAPB-F4PDA COF as an orange color solid in quantitative yield.

COF film synthesis

Preparation for thin films with 0.5 to 3 hours of reaction time

A stock solution of 4MMCA-PDA (24 mg) and TAPB (17 mg) in 0.6 ml of THF was prepared. Films 1-4 was prepared by spin coating with different parameters onto 1.5 cm x 1.5 cm glass substrate. The conditions and thickness were listed in table S1. Before conversion to COF, the rough edges of the films were trimmed off by razor blade.

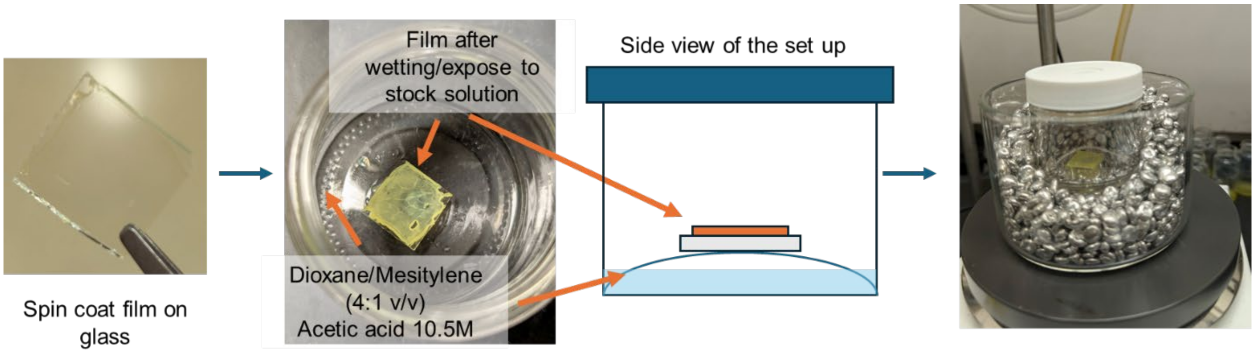

Figure S1. Experiment setup to convert the precursor film to COF film.

Conversion of 4MMCA-PDA/TAPB film to TAPB-PDA COF: the film was wetted with a solution of anhydrous 1,4-dioxane (1.2 ml), mesitylene (0.3 ml), and acetic acid 10.5M (0.7 ml) and placed horizontally on a Teflon holder in a 20ml scintillation vial or placed on top of the concaved surface of a 5.2 cm OD x 5 cm height vial (see Figure S1). The aforementioned solution (0.5 – 1 ml) was placed at the bottom of the vial (Figure S1). The vial was capped and heated to 70 °C overnight or for indicated time. After completion, the COF film was washed by methanol (3 times, 10 ml each) and acetone (3 times, 10 ml each) and stored in acetone until next step.

Activation of COF film using flow of nitrogen method: a COF film was stored in acetone. From acetone, solvent was exchanged to DCM (3 times, 1 hour each) then n-hexane (3 times, 1 hour each) and stored in n-hexane overnight. Hexane was then decant. The vial was then placed under the constant flow of N_2 and was heated to 150 °C during 1.5 hours and kept at 150 °C for 3 hours, followed with cooling down to RT for 30 min and left in house vacuum overnight. This process yielded an activated COF film as yellow color film.

The thickness of the TAPB-PDA COF film after 3 hour conversion was measured by contact profilometer to give the value of 4.58 ±1.06 μm

Table S1. Conditions and thickness for spin coat films

Film #	rpm	Amount of solution	Thickness	Time of conversion to COF
1	10,000	20 μL	2.3 μm	1 h
2	10,000	20 μL	2.2 μm	2 h
3	5,000	20 μL	1.45 μm	0.5 h
4	5,000	30 μL	1.83 μm	3 h

The precursor film for 22 hour conversion

A stock solution of 4MMCA-PDA (10 mg) and TAPB (7mg) in 1 ml THF was prepared. Approximately 0.05 ml solution was blade cast onto a glass slide and allowed to dry before the next step.

Conversion of the film to COF: the film was wetted with a solution of anhydrous 1,4-dioxane (1.2 ml), mesitylene (0.3 ml), and acetic acid 10.5M (0.7 ml) and placed vertically in a Teflon holder in a 20ml scintillation vial. The aforementioned solution (0.5 ml) was placed at the bottom of the vial. The vial was capped and heated to 70 °C overnight. After completion, the COF film was washed by methanol (3 times, 10 ml each) and acetone (3 times, 10 ml each) and stored in acetone until next step. The film was activated using flow of nitrogen method described previously.

COF in designed shape

Casting 2 layers film

A stock solution of 4MMCA-PDA (100 mg) in 1.8 ml THF was prepared. This solution was cast onto a Teflon substrate using a blade coater from MTI Corporation (120 μm thickness, speed 15) for ~ 5 times (~400 μL each time). The film was allowed to dry before casting of the 2nd layer. A stock solution of 4MMCA-PDA (50 mg, 0.15 mmol) and TAPB (32 mg, 0.09 mmol) in 0.9 ml THF was prepared. This solution was cast onto the 1st layer using a blade coater (120 μm thickness, speed 20) ~ 4 times (~200 μL each time). The film was allowed to dry then lift off from the Teflon substrate and transfer to a glass substrate. The film was then laser cut into desired shapes and was a stand alone film after cutting.

Conversion of the film to COF: the glass substrate was wetted with a solution of anhydrous 1,4-dioxane (1.2 ml), mesitylene (0.3 ml), and acetic acid 10.5M (0.7 ml) and placed on top of the concaved surface of a 5.2 cm OD x 5 cm height vial. The 4MMCA-PDA/TAPB precursor film in specific shape was placed on top of the substrate with the surface containing 4MMCA-PDA/TAPB layer was up. The aforementioned solution (~ 1 ml) was placed at the bottom of the vial. The vial was capped and heated to 70 °C overnight. After completion, the COF film was washed by methanol (3 times, 10 ml each) and acetone (3 times, 10 ml each) and stored in acetone until next step. The film was activated using flow of nitrogen method described previously. During activation, the COF with specific shape was sandwiched between 2 glass slides.

Stand alone thick COF film

4MMCA-PDA + TAPB film casting

To a 4 ml vial was added TAPB (34 mg, 0.097 mmol, 1 equivalent) and 4MMCA-PDA (66 mg, 0.196 mmol, 2 equivalent) and 0.6 ml THF. The resulting solution was sonicated to completely dissolve the reagents. The solution was casted onto a 30 mm x 36 mm glass slide (the glass slide was previously cleaned with detergent solution, DI water, EtOH, Acetone and dry in oven). The wet film on glass substrate was covered

with a petri-dish and left in the oven under the flow of nitrogen to ensure slow evaporation of THF for a smooth film. In this experiment, the film was left in the oven with constant N_2 flow overnight, but it is recommended to leave the film for at least 2 days to ensure the complete evaporation of THF. The film was then transferred to vacuum oven and was placed under vacuum and heating at 70 ºC for 4 hours followed by cooling down to RT and staying under vacuum overnight.

Convert film to COF

The precursor film prepared above was placed on top of the concaved surface of a 5.2 cm OD x 5 cm height vial. The solution of 1,4-dioxane, mesitylene, and acetic acid 10.5M (1.2:0.3:0.7 v/v/v, ~ 1 ml) was placed at the bottom of the vial (Figure S1). The vial was capped and heated to 70 °C overnight. After completion, the COF film was washed by methanol (3 times, 10 ml each) and acetone (3 times, 10 ml each) and stored in acetone until next step. The film was activated using flow of nitrogen method described previously.

Aerosol jet printing

Aerosol jet printing of 4MMCA-PDA/TAPB precursor mixture: for AJP, the substrates are mico 90 cleaned corning glass. Before printing, the substrates was ozone-plasma treated for at least 20 mins. A stock solution composing of 10 mg 4MMCA-PDA polyimine and 6 mg of TAPB in 5ml chloroform/terpineol (9:1 by weight) was used for printing. AJ300-UP (Optomec) with 300 μm nozzle and 3/32" ID tubing were used for printing. The ultrasonic atomizer flow rate was set at 35 – 50 sccm, the sheath gas flow rate was at 50 sccm. The stage temperature was set to 60 °C and the atomizer bath was set as 35 °C, the atomizer current was set to maxium level. Printing speed was at 4 mm/s and the number of passes was 32.

After printing, the precursor film was placed in the oven at 60 °C under house vacuum for 3 hours and leave under vacuum without heating overnight. The temperature was brought up to 70 °C and hold for 3 hours before turinng off the heat and left in vacuum over night.

Conversion of the film to COF: The printed precursor film was wetted with a solution of anhydrous 1,4-dioxane (1.2 ml), mesitylene (0.3 ml), and acetic acid 10.5M (0.7 ml) and placed on top of the concaved surface of a 5.2 cm OD x 5 cm height vial. The aforementioned solution (~ 1 ml) was placed at the bottom of the vial. The vial was capped and heated to 70 °C overnight. After completion, the COF film was washed by methanol (3 times, 10 ml each) and acetone (3 times, 10 ml each) and stored in acetone until next step. The film was activated using flow of nitrogen method described previously.

COF/CNT composite

Three stock solutions of CNT in anhydrous THF were prepared:

Solution 1: 2.1 mg CNT in 26 ml THF

Solution 2: 2.4 mg CNT in 6 ml THF

Solutions 3: 2.6 mg CNT in 3.2 ml THF

Resulting mixture was sonicated for 30 mins.

Add 1 ml of each solutions to a solid mixture of TAPB (3.5 mg) and 4MMCA-PDA (5 mg) to form 3 stock solutions: solution 1 gave 0.9 wt% CNT, solution 2 gave 4.5 wt% CNT, and solution 3 gave 8.7% wt CNT. The solutions were sonicated for 30 mins. Each solutions was casted into 2 films on the 2.5 cm x 2.5 cm glass substrate using slow evaporation casting technique under the flow of nitrogen.

Synthesis of COF/CNT composite: the CNT/precursor film was placed on top of the concaved surface of a 5.2 cm OD x 5 cm height vial. The solution of 1,4-dioxane, mesitylene, and acetic acid 10.5M (1.2:0.3:0.7 v/v/v, ~ 1 ml) was placed at the bottom of the vial. The vial was capped, placed at RT for 1 hour and heated to 70 °C overnight. After completion, the COF film was washed by methanol (3 times, 10 ml each) and acetone (3 times, 10 ml each) and stored in acetone until next step.

Activation of COF film using flow of nitrogen method: COF/CNT composite in acetone was exchanged to DCM and n-hexane. n-Hexane was decanted out of the vial and the vial was then placed under the constant flow of N_2 and was heated to 150 °C for 1.5 hours and kept at 150 °C for 3 hours, followed with cooling down to RT for 30 mins and left under house vacuum overnight. This process yielded an activated TAPB-PDA COF/CNT composite.

Fourier Transform Infrared Spectroscopy

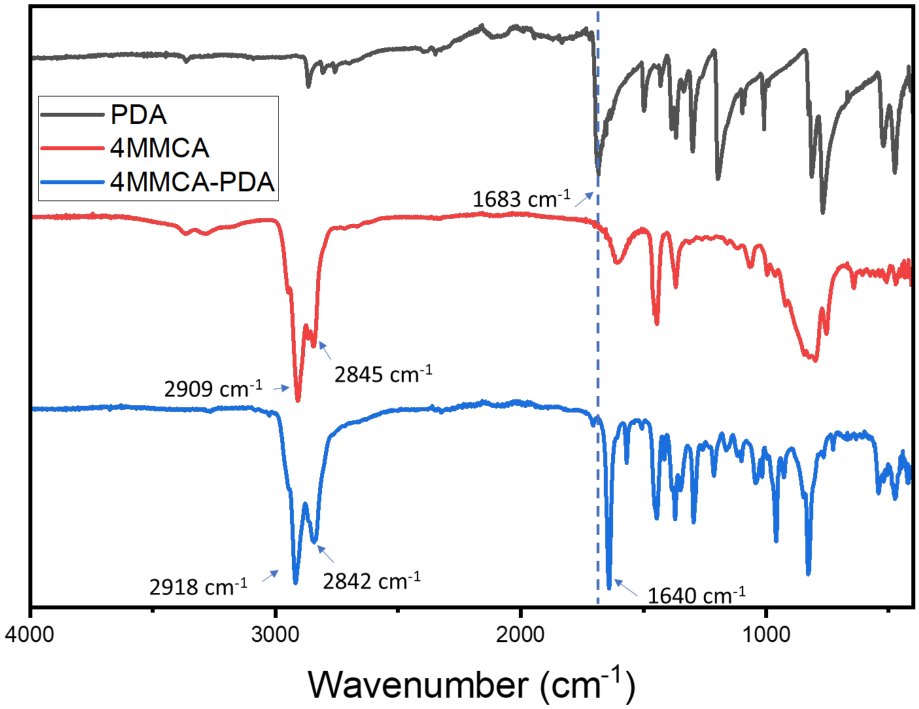

Figure S2. FTIR spectra of PDA, 4MMCA and 4MMCA-PDA polyimine.

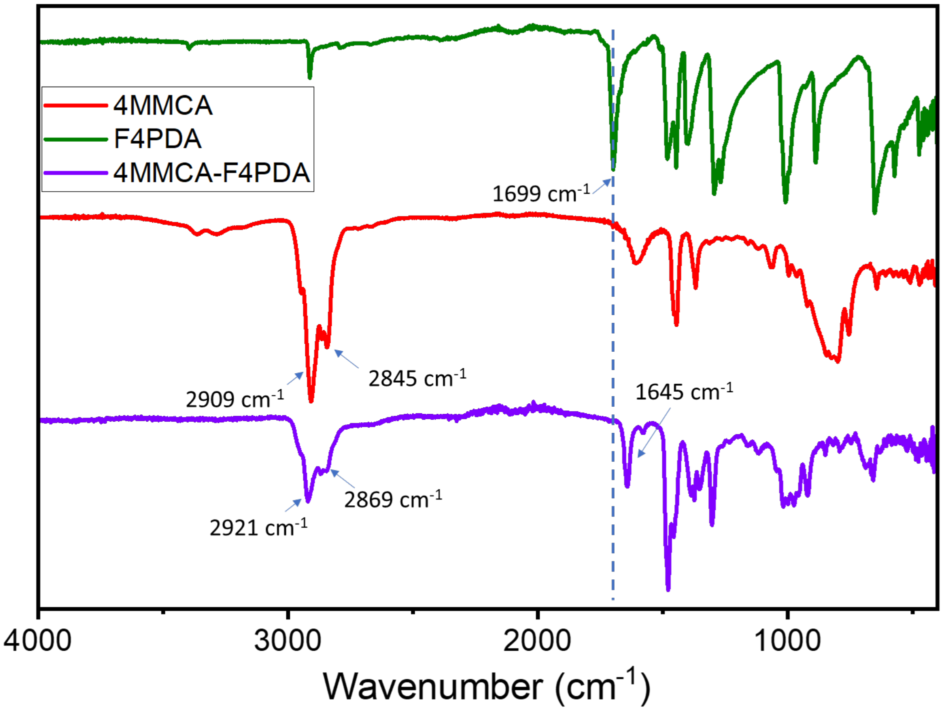

Figure S3. FTIR spectra of F4PDA, 4MMCA, and 4MMCA-F4PDA polyimine.

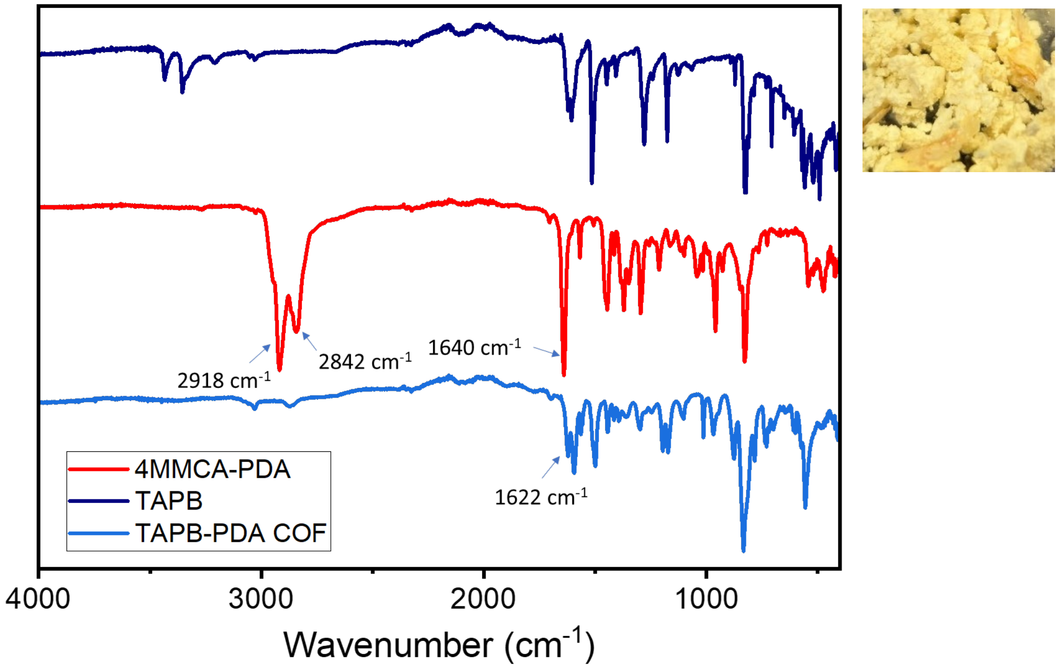

Figure S4. FTIR spectra of TAPB, 4MMCA-PDA polyimine and TAPB-PDA COF and a picture of TAPB-PDA COF powder

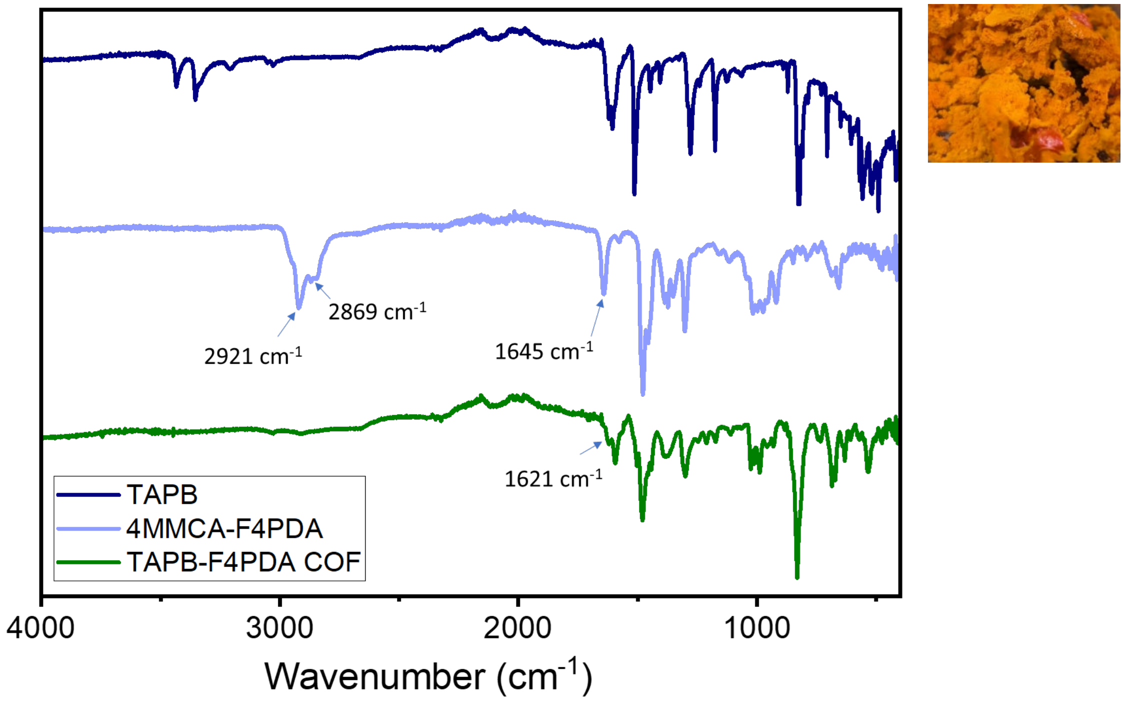

Figure S5. FTIR spectra of TAPB, 4MMCA-F4PDA, and TAPB-F4PDA COF and a picture of TAPB-F4PDA COF

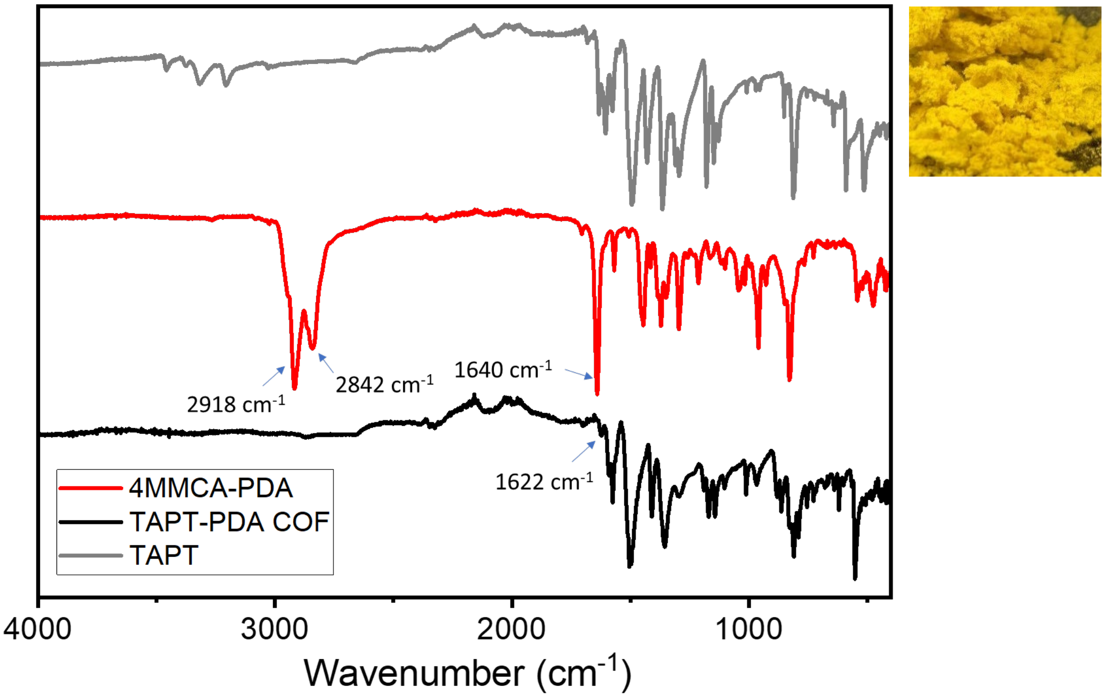

Figure S6. FTIR spectra of TAPT, 4MMCA-PDA, and TAPT-PDA COF and a picture of TAPT-PDA COF

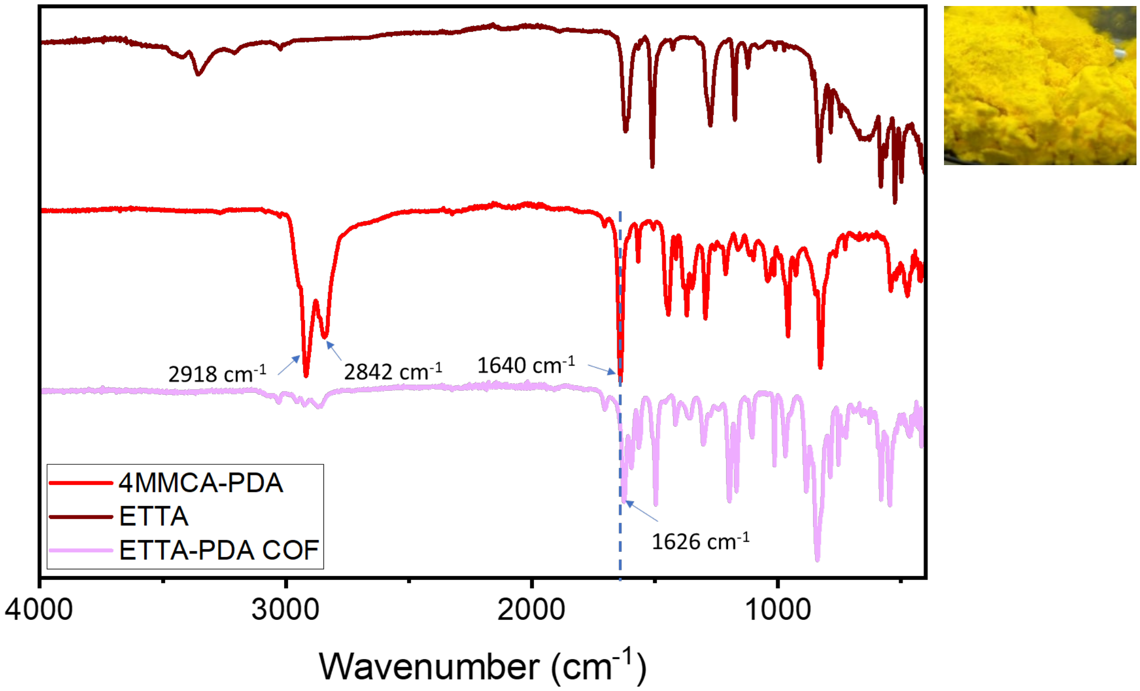

Figure S7. FTIR spectra of ETTA, 4MMCA-PDA, and ETTA-PDA COF and a picture of ETTA-PDA COF

Nuclear Magnetic Resonance Spectroscopy

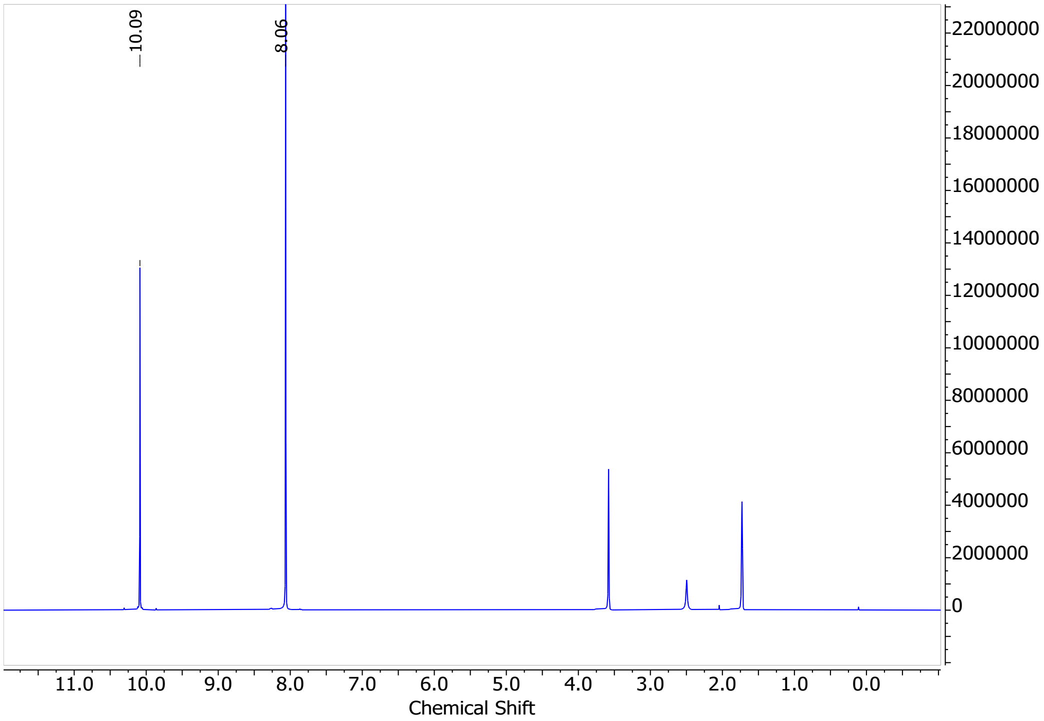

Figure S8. ¹H NMR of PDA in THF-D$_8$

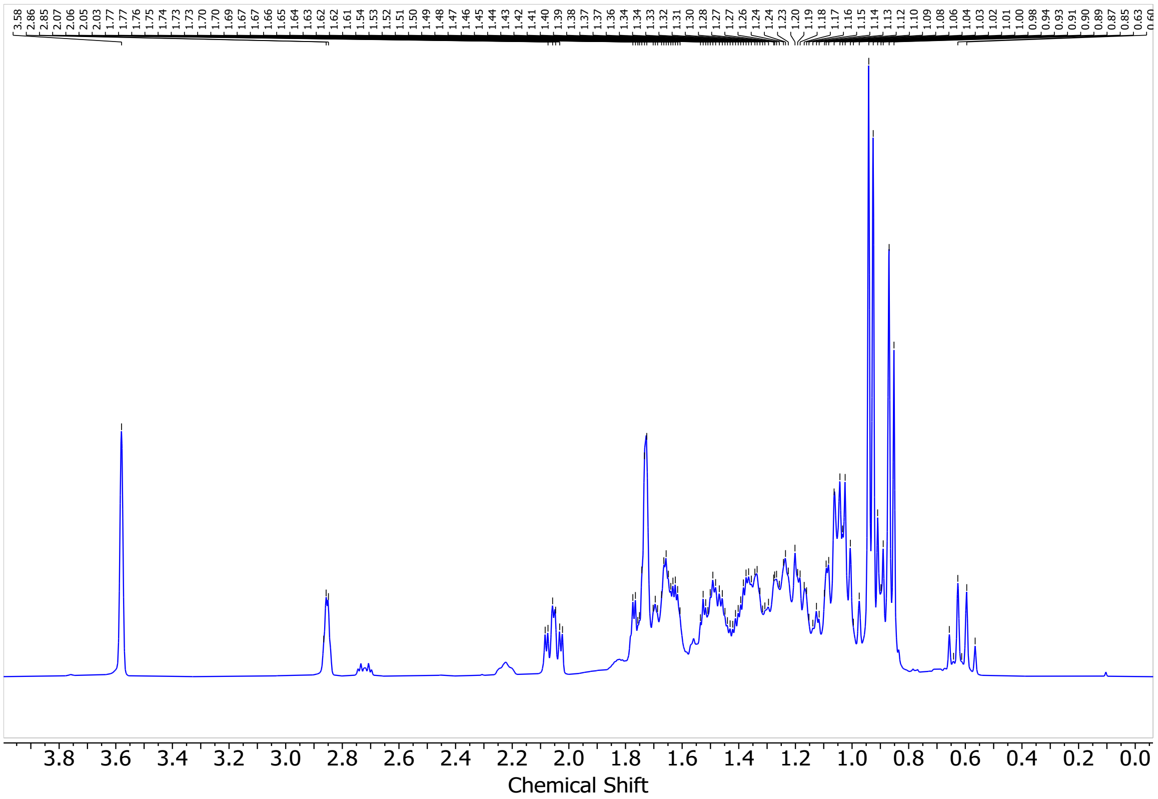

Figure S9. ¹H NMR of 4MMCA in THF-D$_8$

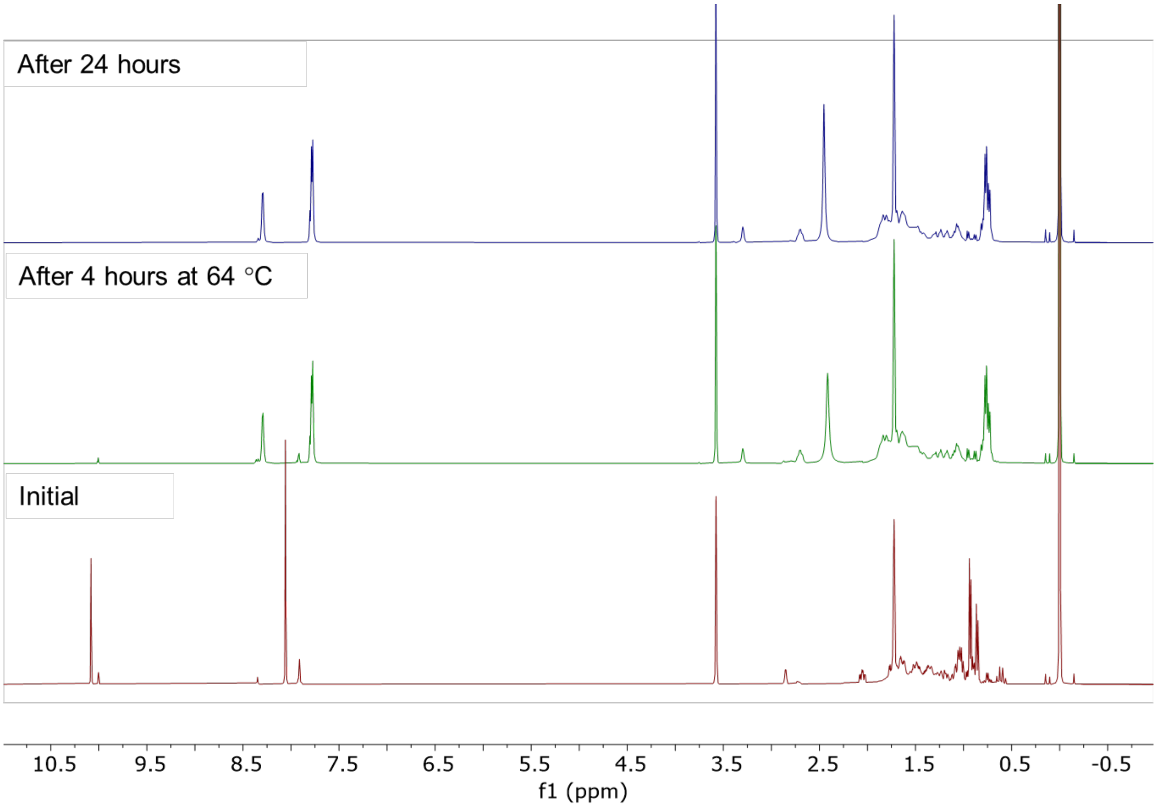

Figure S10. ^{1}H NMR of reaction of 4MMCA and PDA in THF-D_8

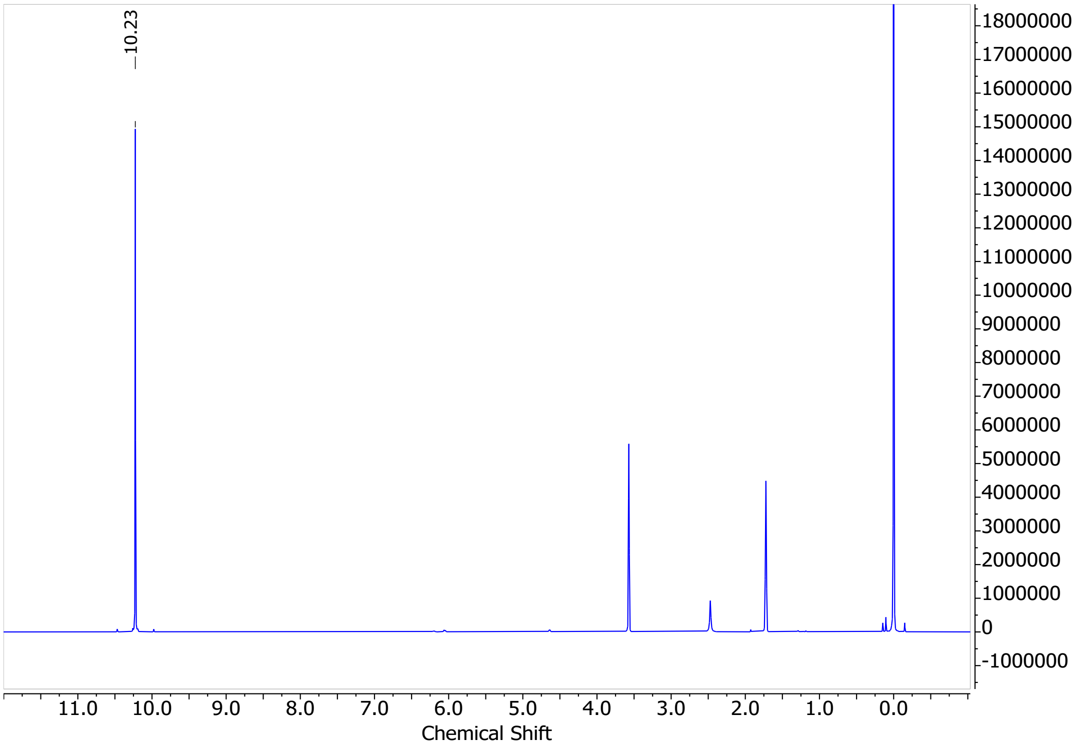

Figure S11. ^{1}H NMR of F4PDA in THF-D_8

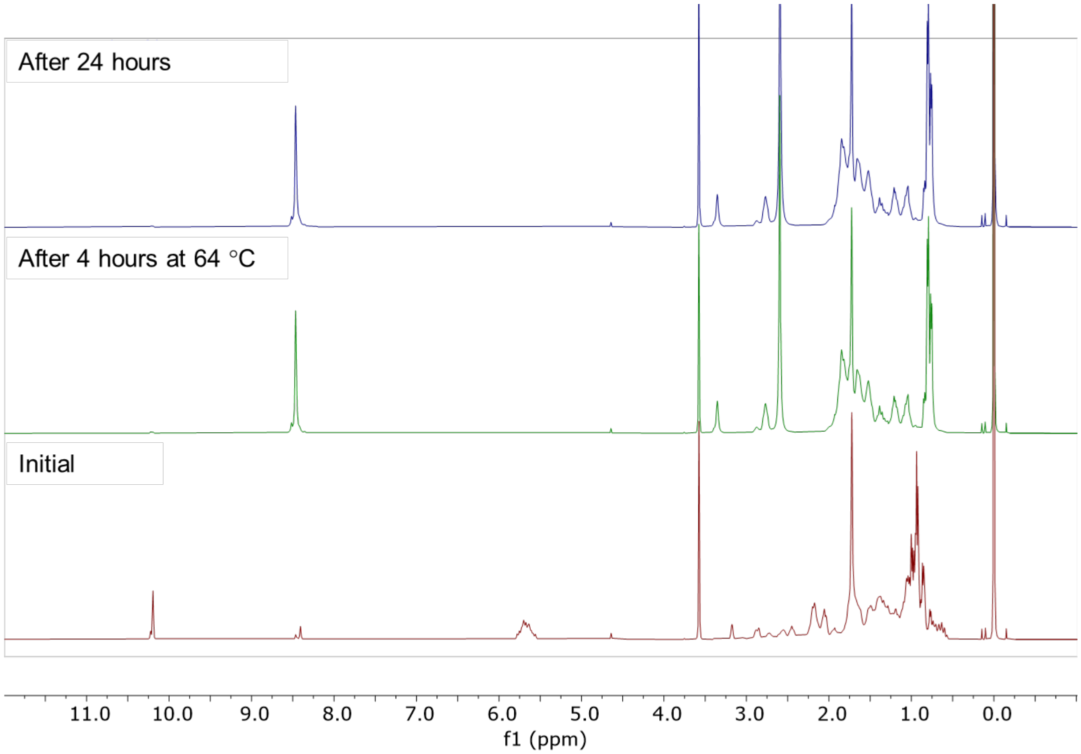

Figure S12. ^{1}H NMR of reaction of 4MMCA and F4PDA in THF-D_8

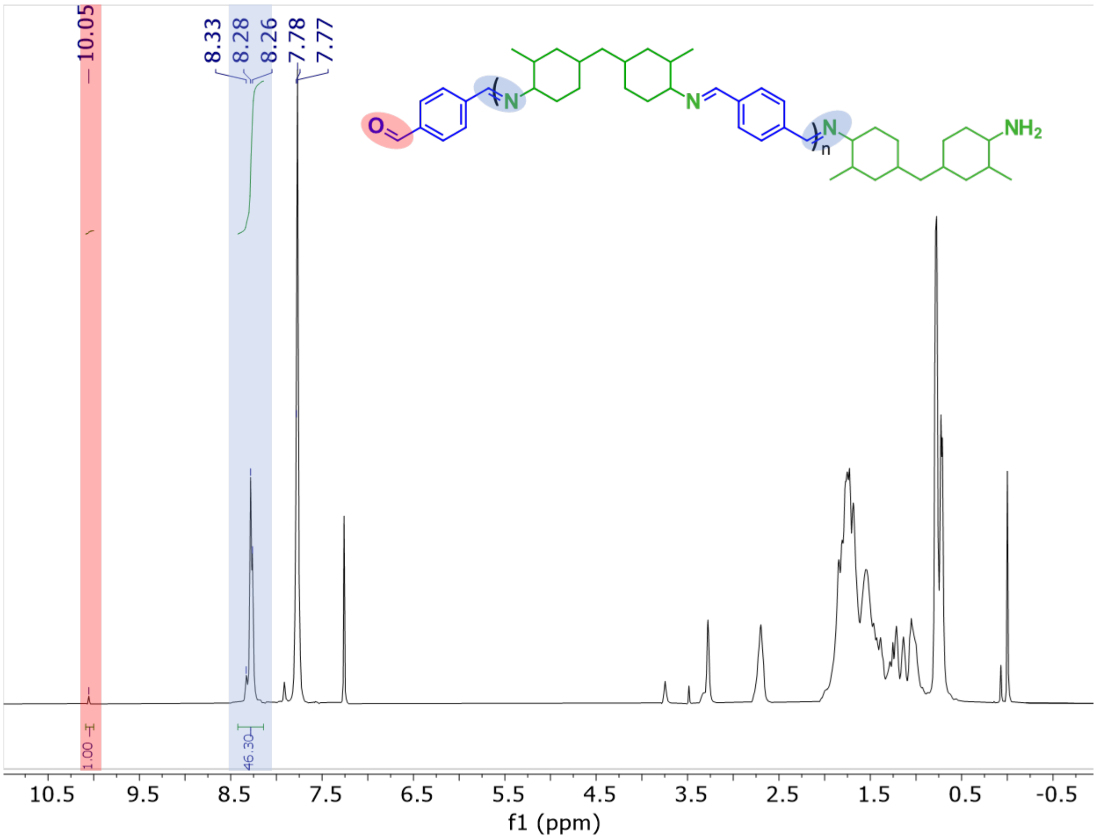

Figure S13. ^{1}H NMR spectra of 4MMCA-PDA polyimine in $CDCl_3$

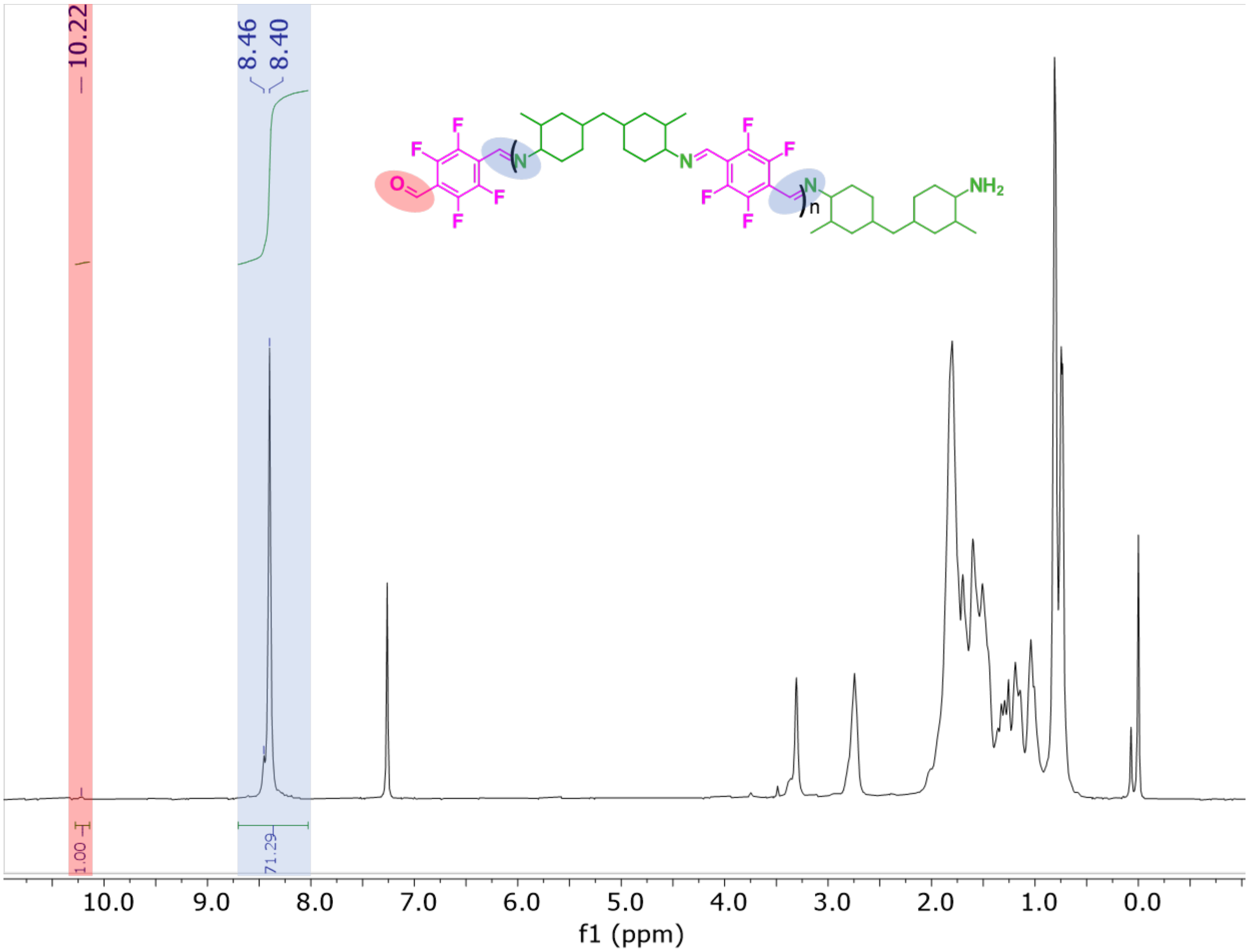

Figure S14. ^{1}H NMR spectra of 4MMCA-F4PDA polyimine in $CDCl_3$

Based on the Carothers equation

$$\bar{x}_n = \frac{n_o}{n} = \frac{1}{1-p}$$

Where n_o is the initial amount of monomer units and n is the number of polymer molecules, the number average degree of polymerization (DP or $\bar{x}_n$) can be calculated where p is the conversion, or extent of the reaction, which represents the fraction of the functional groups that have undergone reaction at a time of interest.[3]

After polymerization and isolation of the polyimine, we use this equation to calculate the DP and molecular weight of the isolated/purified polymer using ^{1}H NMR.

4MMCA-PDA.

$$p = \frac{46.3}{(1+46.3)} = 0.979$$

$$\text{DP} = \frac{1}{(1-0.979)} = 47.62$$

Molecular weight = DP x (molecular weight of repeat unit) = 47.62 × (336.52 g/mol) = 16,025 g/mol

4MMCA-F4PDA.

$$p = \frac{71.3}{(1+71.3)} = 0.986$$

$$\mathrm{DP} = \frac{1}{(1-0.986)} = 72.43$$

Molecular weight = DP x (molecular weight of repeat unit) = 72.43 × (408.48 g/mol) = 29,177 g/mol

NMR of hydrolyzed TAPB-PDA COF made from 4MMCA-PDA and TAPB

Scheme S2. Hydrolysis of TAPB-PDA COF, made from 4MMCA-PDA and TAPB, in powder form or film in DCl/DMSO-D_6

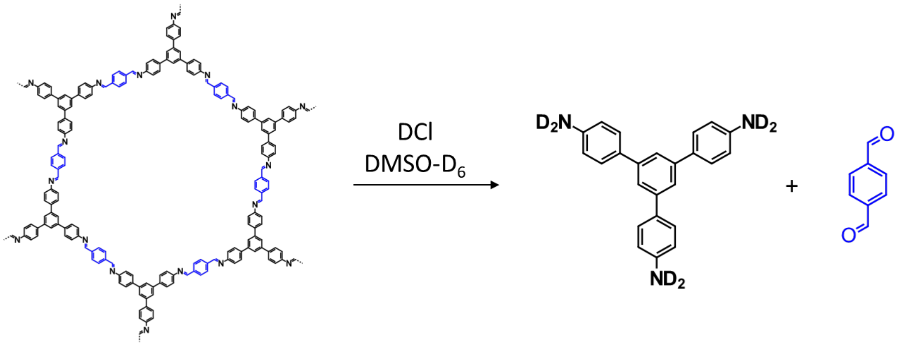

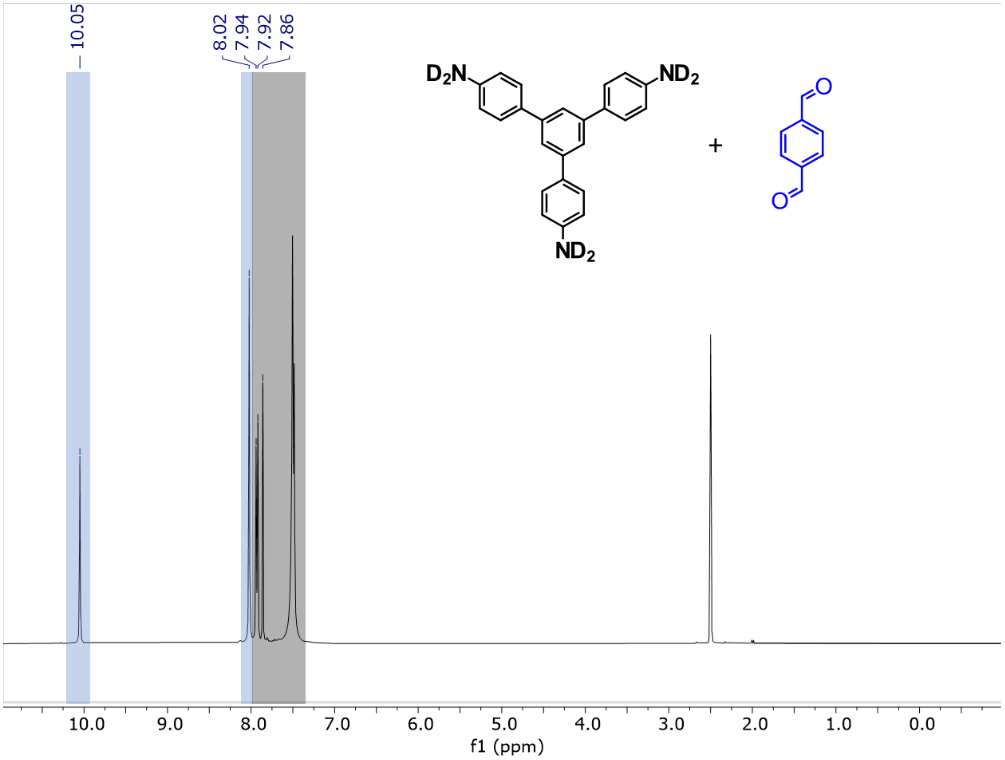

Figure S15. ^{1}H NMR spectra of TAPB-PDA COF powder, made from 4MMCA-PDA and TAPB, hydrolyzed in DMSO-D_6/DCl. The peaks in blue regions are PDA peaks and peaks in black regions are TAPB peaks with the peaks between 7.6 to 7.4 ppm are the overlapping of DCl/DMSO-D_6 and aryl C-H peaks of TAPB.

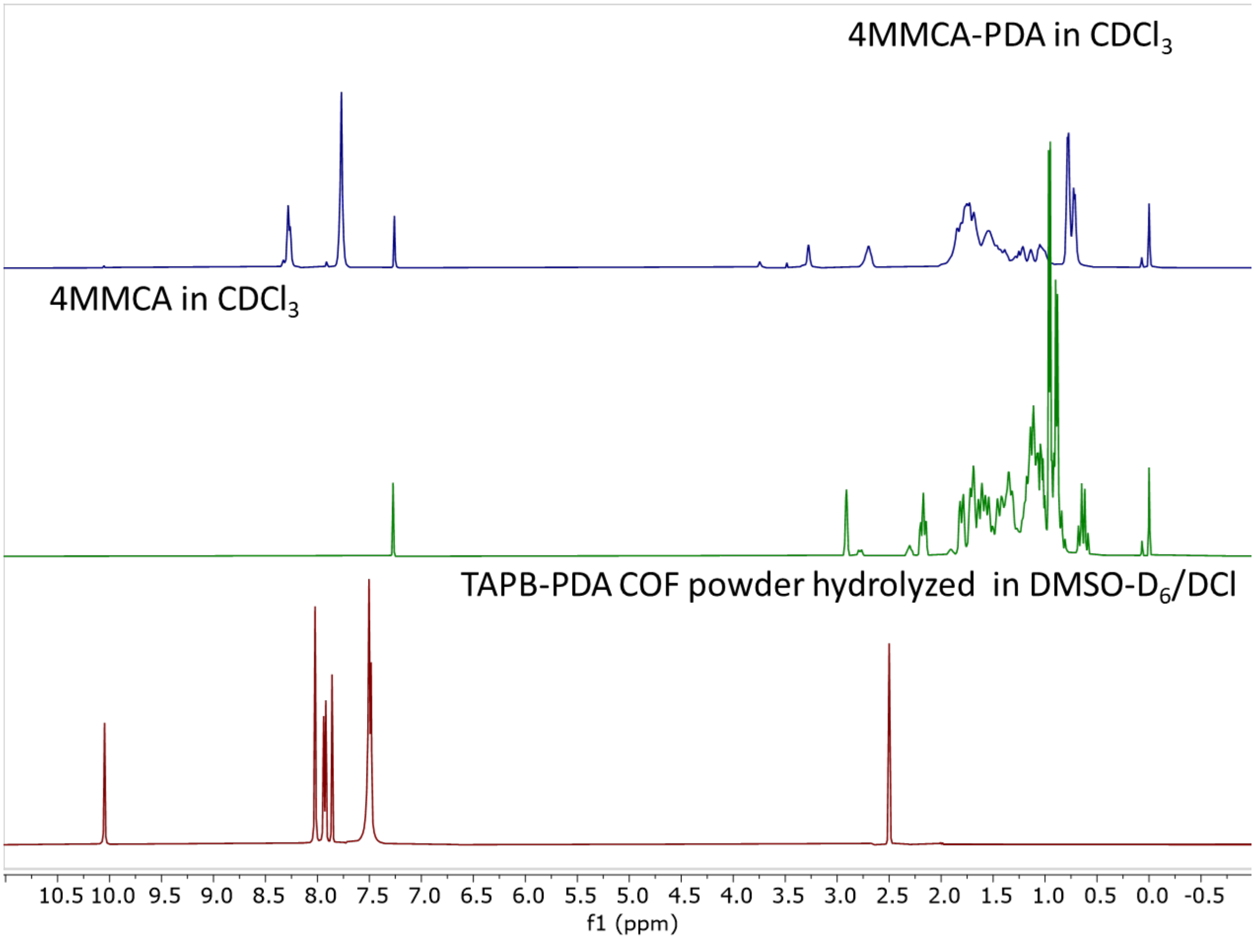

Figure S16. Stacking of ^{1}H NMR spectra of 4MMCA-PDA in $CDCl_3$ (top, blue), 4MMCA in $CDCl_3$ (middle, green), and TAPB-PDA, made from 4MMCA-PDA and TAPB, hydrolyzed in DCl/DMSO-D_6. The missing of peaks in the chemical shift ranges of 3.0 to 0.5 ppm indicates no 4MMCA left in the final COF product.

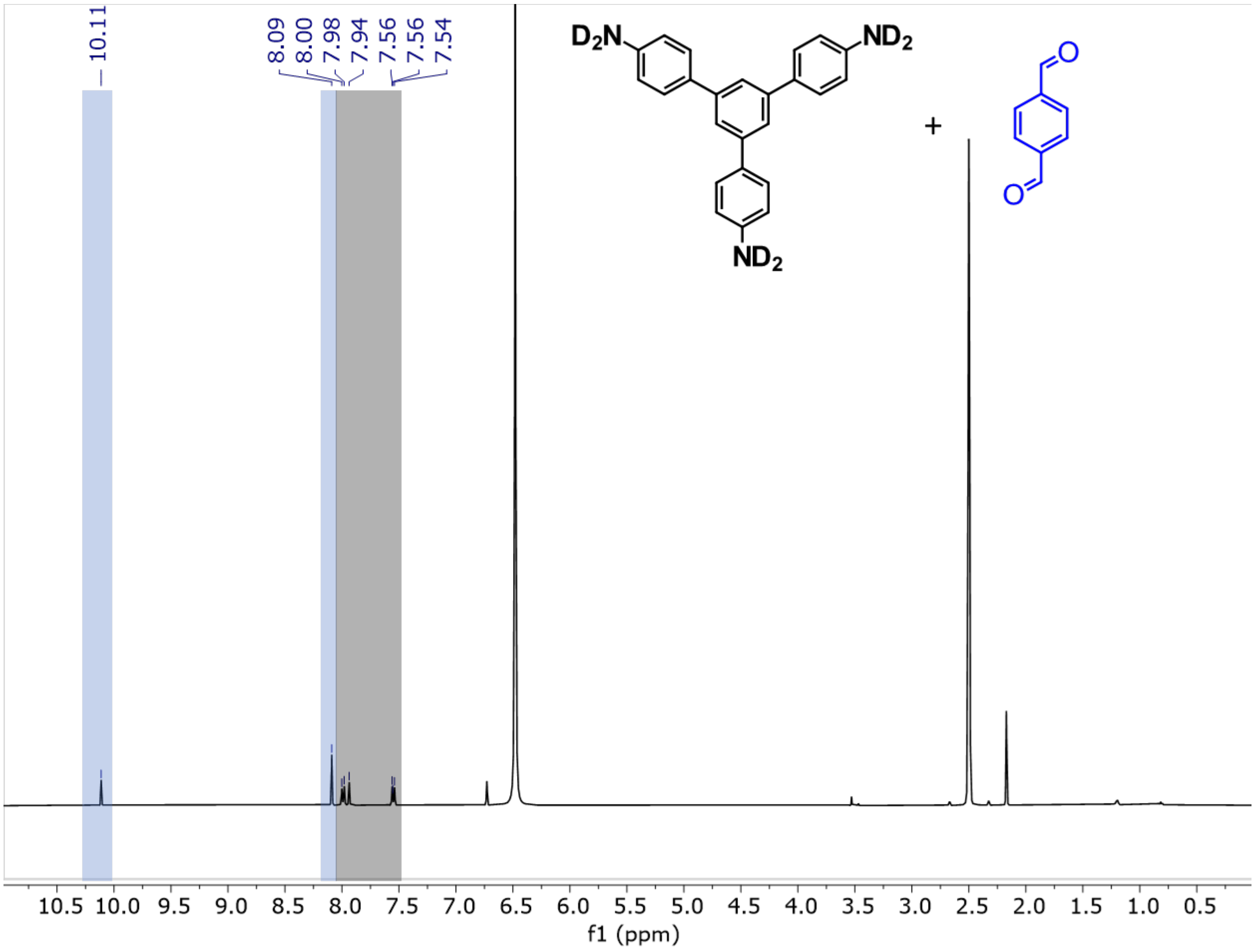

Figure S17. ^{1}H NMR of TAPB-PDA COF film, made from 4MMCA-PDA and TAPB through 2-layer casting procedure, hydrolyzed in DMSO-D_6/DCl. The peaks in blue regions are PDA peaks and peaks in black regions are TAPB peaks.

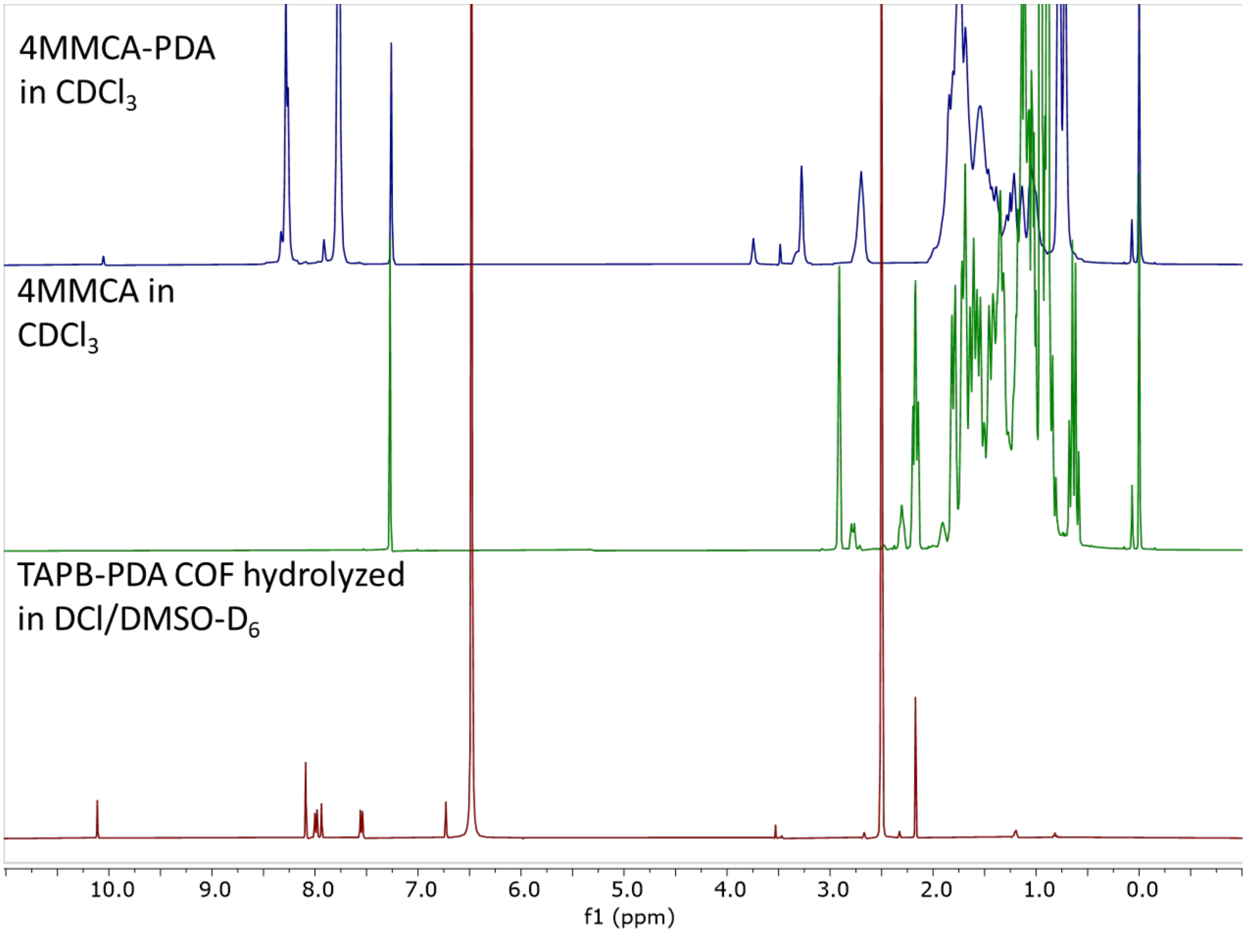

Figure S18. Stacking of ^{1}H NMR spectra of 4MMCA-PDA in $CDCl_3$ (top, blue), 4MMCA in $CDCl_3$ (middle, green), and TAPB-PDA film, made from 4MMCA-PDA and TAPB, hydrolyzed in DCl/DMSO-D_6. The missing of peaks in the chemical shift ranges of 3.0 to 0.5 ppm indicates no 4MMCA left in the final COF product.

Gel Permeation Chromatography (GPC)

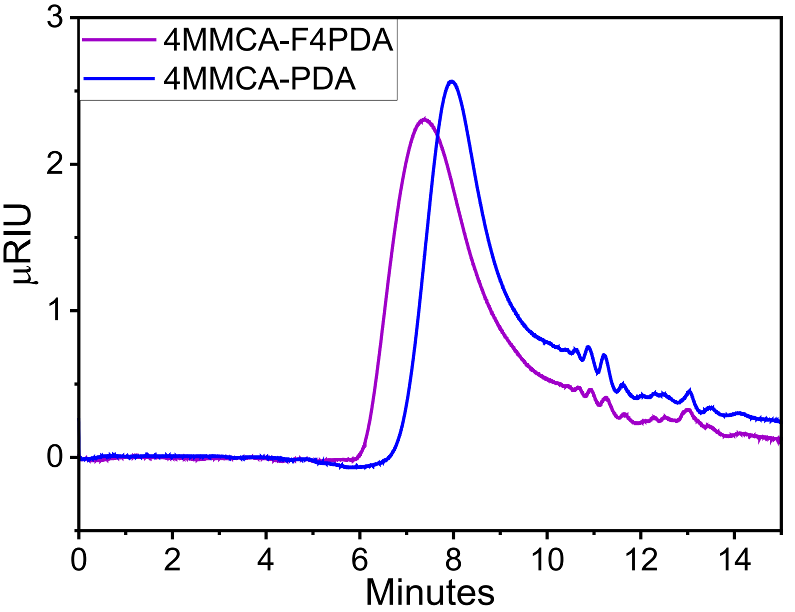

Figure S19. GPC traces of 4MMCA-PDA and 4MMCA-F4PDA polyimines

Thermogravimetric Analysis (TGA)

For the 4MMCA-PDA, 5.529 mg of sample material was used. The weight lost vs. temperature is shown in Figure S20. The $T_{d,1\%}$ is 319.90 °C and the $T_{d,5\%}$ is 372.80 °C.

For the 4MMCA-F4PDA, 4.658 mg of sample material was used. The weight lost vs. temperature is shown in Figure S21. The $T_{d,1\%}$ is 329.78 °C and the $T_{d,5\%}$ is 362.48 °C.

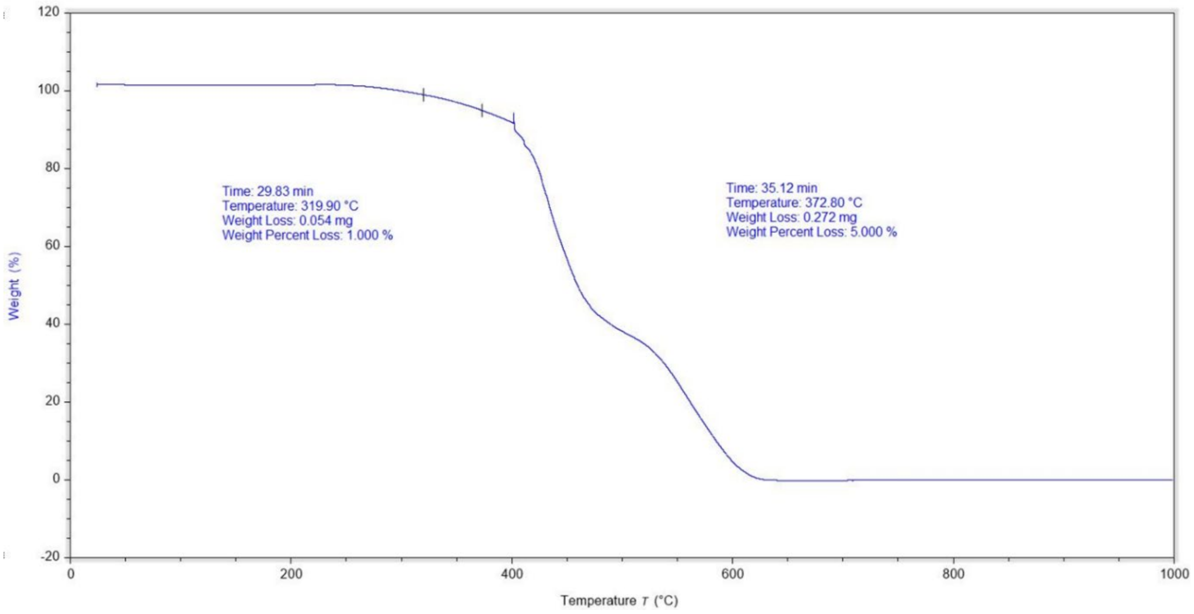

Figure S20. TGA of 4MMCA-PDA polyimine with 1% mass loss at 319.9 °C and 5% mass loss at 372.8 °C

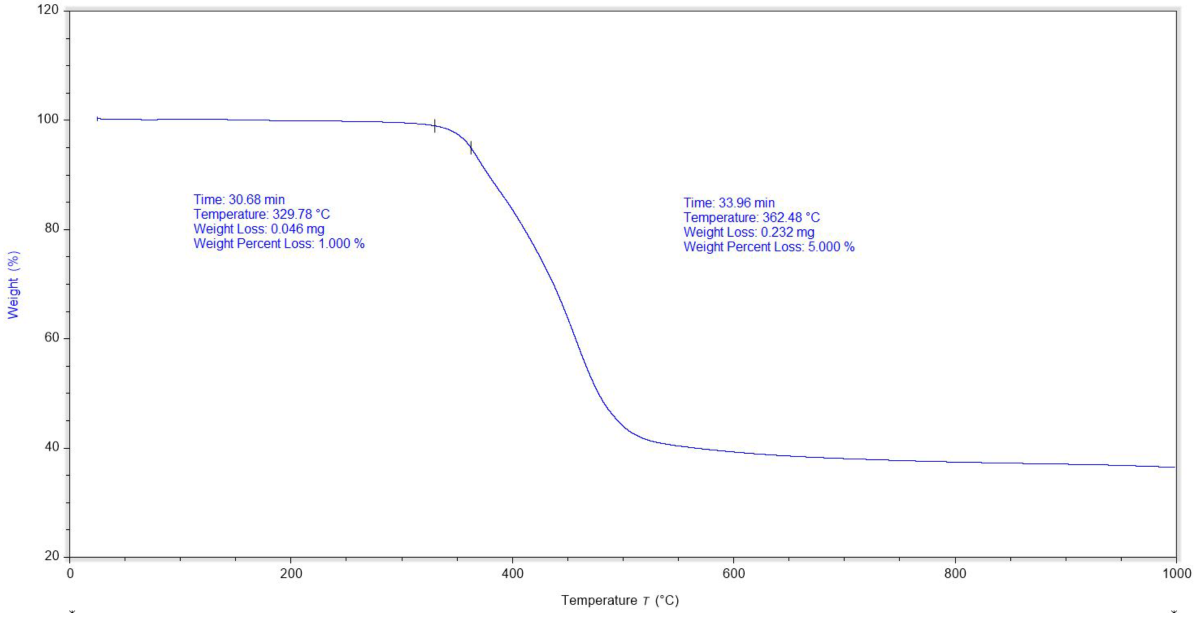

Figure S21. TGA of 4MMCA-F4PDA polyimine with 1% mass loss at 329.78 °C and 5% mass loss at 362.48 °C

Differential Scanning Calorimetry (DSC)

The final Heat run plot was used to determine T_g. For 4MMCA-PDA polyimine (Figure S22) its T_g is 190.37 °C. For 4MMCA-F4PDA polyimine (Figure S23) its T_g is 173.08 °C.

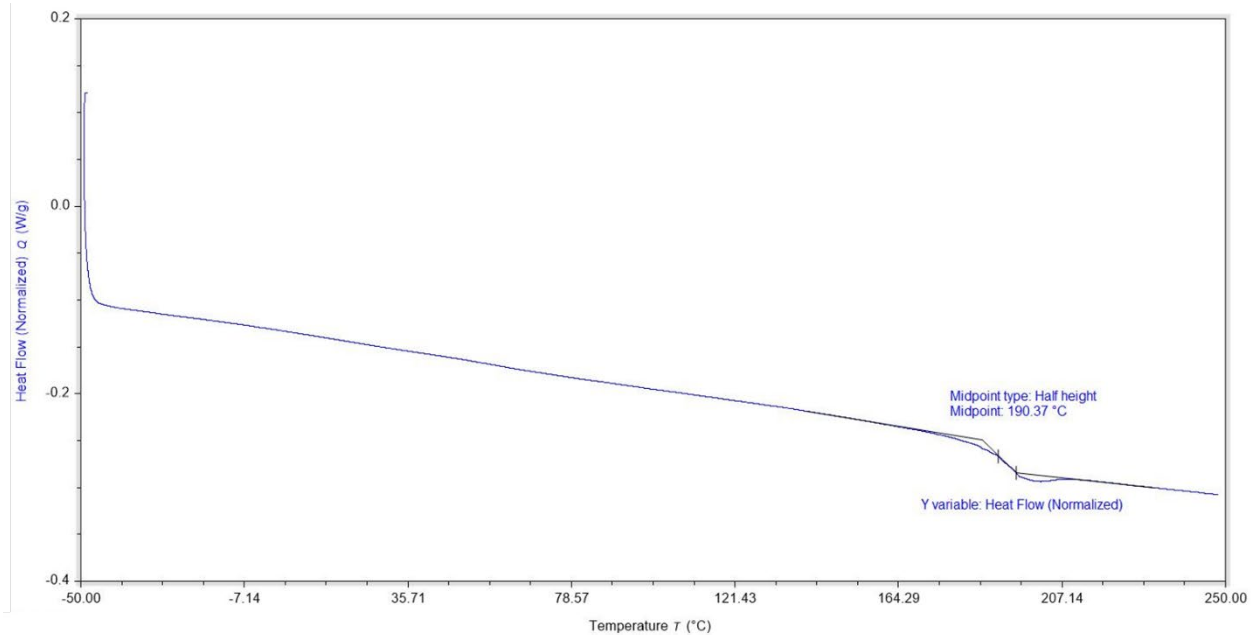

Figure S22. DSC of 4MMCA-PDA polyimine

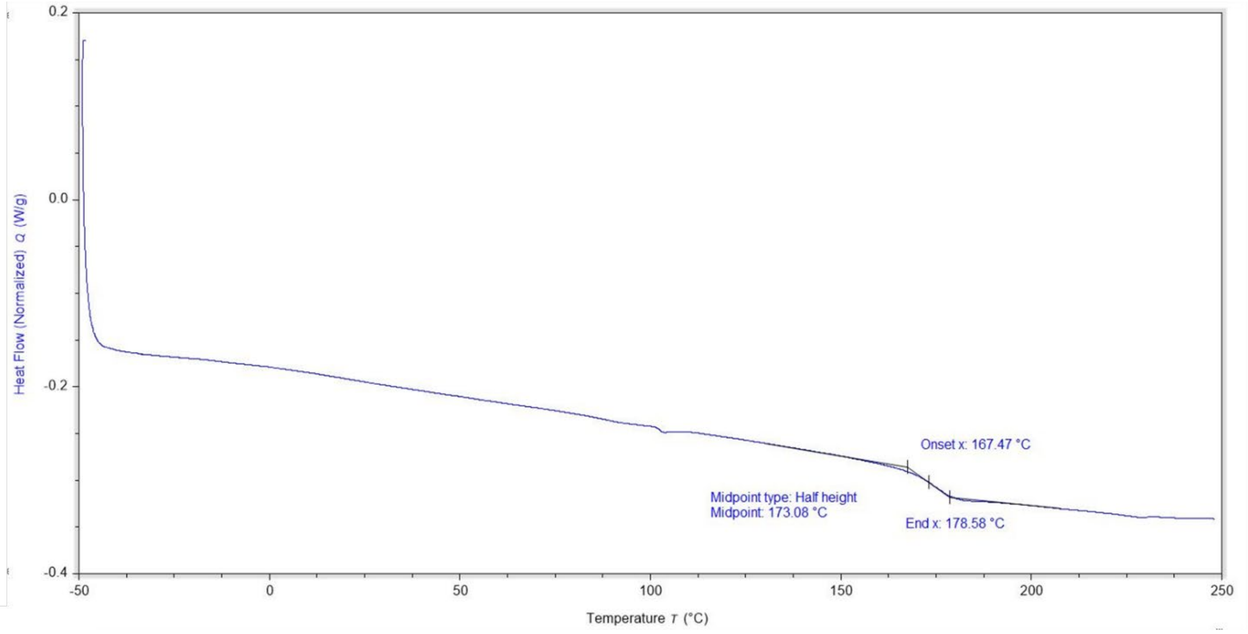

Figure S23. DSC analysis of 4MMCA-F4PDA polyimine

Stress-Strain Analysis

Three samples were tested for 4MMCA-PDA (Figure S24), and the derived mechanical properties are presented in Table S2. Six samples were tested for 4MMCA-F4PDA (Figure S25), and the derived mechanical properties are presented in Table S3. The results show that the elastic modulus, ultimate tensile strength, and fracture are comparable, with 4MMCA-F4PDA being slightly higher for each property. In general, the 4MMCA-F4PDA has slightly better mechanical properties than 4MMCA-PDA.

Table S2. Derived mechanical properties of 4MMCA-PDA.

4MMCA-PDA			
Sample #	Elastic Modulus (MPa)	UTS (MPa)	Fracture Strain (%)
1	1239.80	46.69	20.80
2	1113.90	85.14	56.09
3	1449.30	84.15	38.51
Average	1267.67	71.99	38.47
St. Dev.	138.34	17.90	14.40

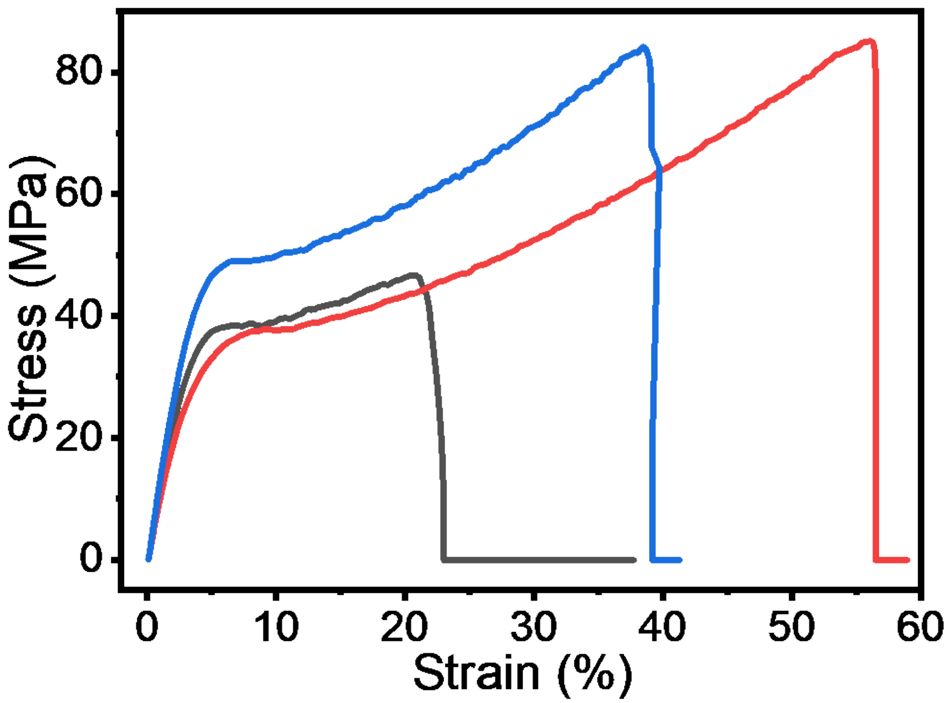

Figure S24. Stress-strain curves of 4MMCA-PDA

Table S3. Derived mechanical properties of 4MMCA-F4PDA

4MMCA-F4PDA

Sample #	Elastic Modulus (MPa)	UTS (MPa)	Fracture Strain (%)
1	908.31	81.49	53.56
2	1451.92	79.65	35.93
3	1994.00	127.07	42.21
4	1477.76	108.76	49.33
5	1633.30	91.19	37.88
6	1661.36	94.87	37.93
Average	1521.11	97.17	42.81
St. Dev.	326.11	16.44	6.50

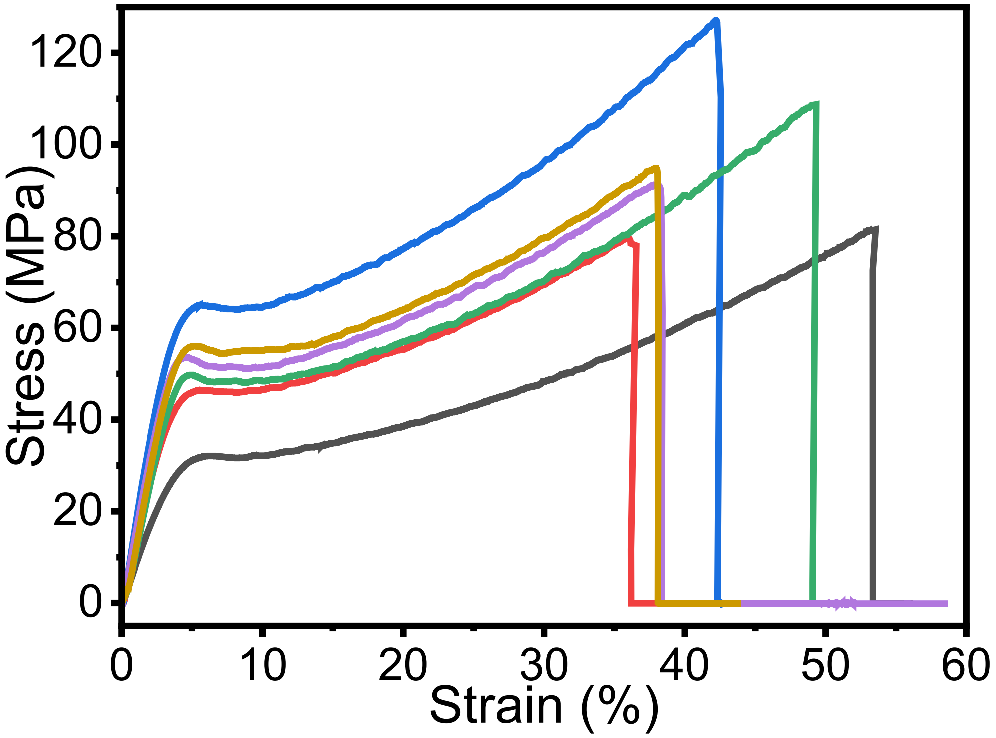

Figure S25. Stress-strain curves of 4MMCA-F4PDA

X-ray Diffraction

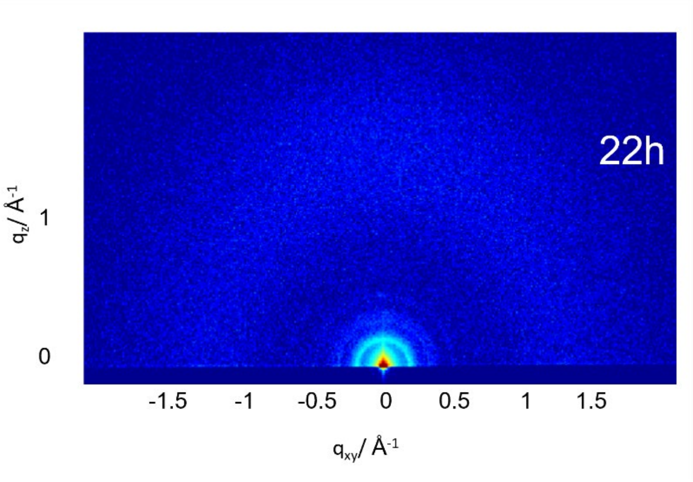

Figure S26. GIWAXS patterns of 4MMCA-PDA COF film made from 4MMCA-PDA and TAPB after 22 hours of heating.

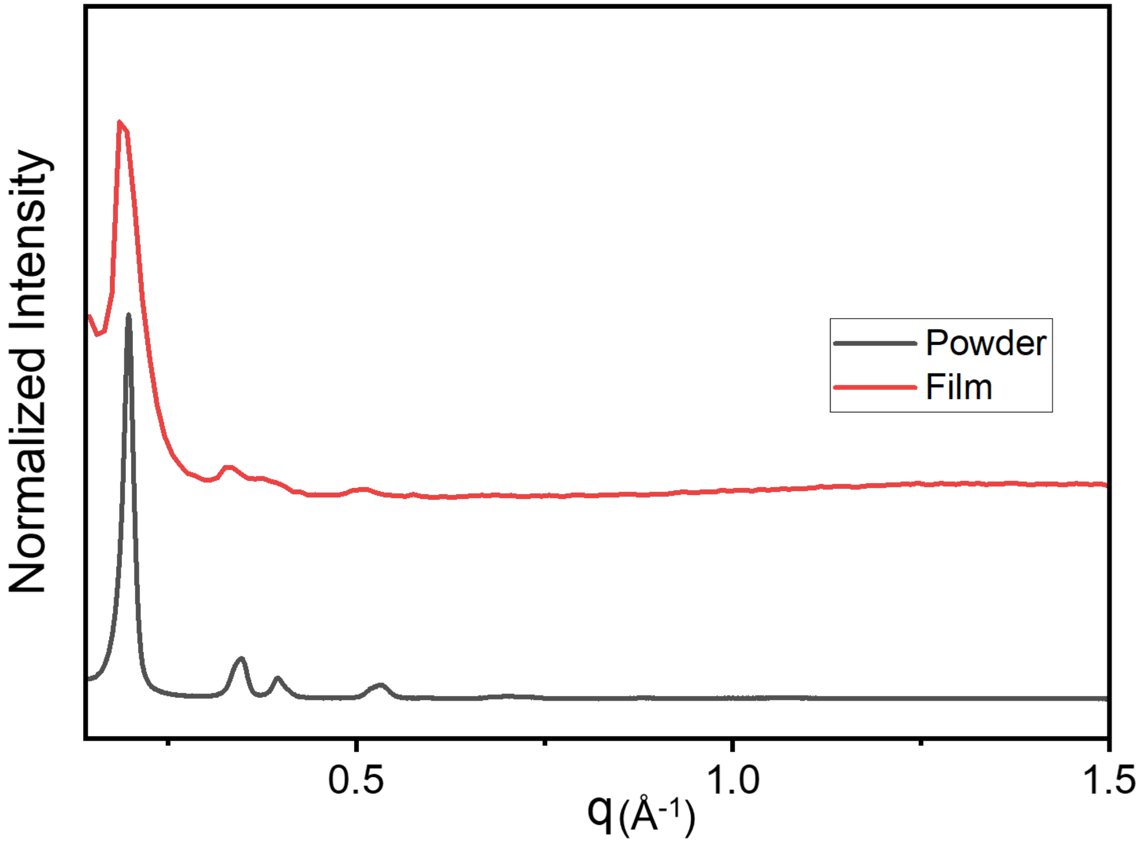

Figure S27. PXRD pattern of TAPB-PDA COF made from 4MMCA-PDA and TAPB in powder form (black) and 1D projection of GIWAXS pattern of TAPB-PDA COF film prepared from 4MMCA-PDA/TAPB precursor film (red)

Crystallite size analysis

The Scherrer's formula was used to compare crystallite size of COFs synthesized from different conditions (temperature or starting materials)[4]

$$t = \frac{K\lambda}{B\cos\theta_B}$$

Where t: crystallite size

λ: X-ray wavelength

K: can have a value of 0.9 or 1 depending on the shapes of the crystallites

θ_B: Bragg angle

B: Full Width Half Max of the Bragg peak

With the same type of COF, the value of λ, K, $\cos\theta_B$ are the same. We can derive the relationship below for crystallite size and the FWHM of the Bragg peak:

$$t \sim \frac{1}{B}$$

Thus, smaller FWHM indicates larger crystallite size and vice versa.

PXRD analysis of TAPB-PDA COF synthesized at RT and with heating at 70 ºC

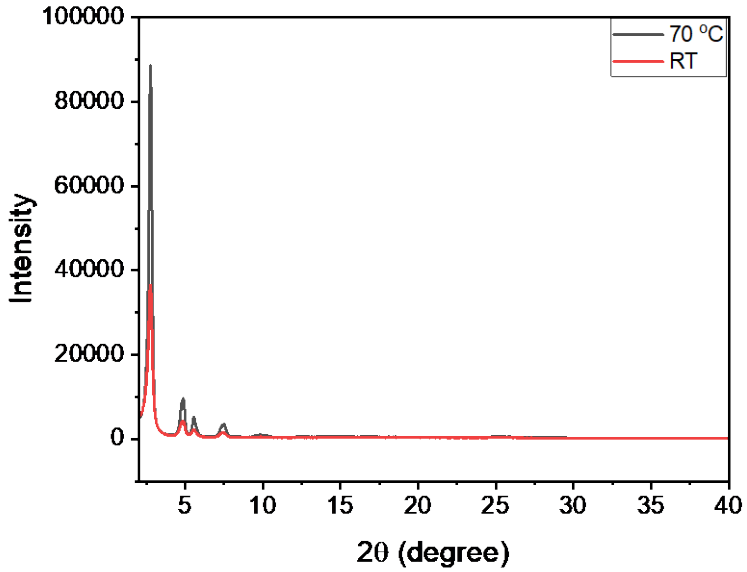

Figure S28. PXRDs of TAPB-PDA COF synthesized at RT and with heating at 70 ºC. Full Width Half Max (FWHM) analysis of Bragg peak at 2.77º gave the value of 0.35 and 0.27 for COF synthesized at RT and 70 ºC, respectively. Result indicates that heating at 70 ºC is needed to generate COF with higher crystallinity and larger crystal domain size.

PXRD analysis of COF synthesized from 4MMCA polyimine and from aldehyde

TAPB-PDA COF

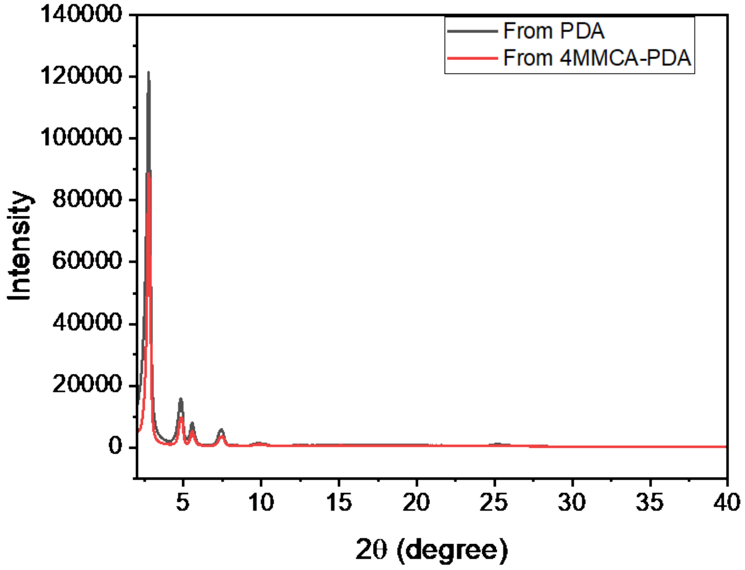

Figure S29. PXRDs of TAPB-PDA COF synthesized from PDA and from 4MMCA-PDA. FWHM analysis of the Bragg peak at 2.77° gave the value of 0.27 and 0.35 for COF synthesized from 4MMCA-PDA and PDA, respectively. The FWHM analysis indicated that the COF made from 4MMCA-PDA polyimine has better crystallinity with larger crystalline domain size.

ETTA-PDA COF

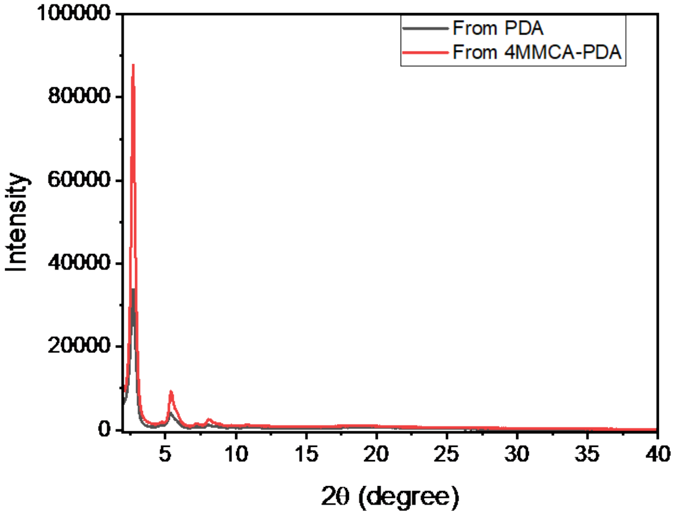

Figure S30. PXRDs of ETTA-PDA COF synthesized from PDA and from 4MMCA-PDA. FWHM analysis of the Bragg peak at 2.72° gave the value of 0.36 and 0.43 for COF synthesized from 4MMCA-PDA and PDA, respectively. The FWHM analysis indicated that the COF made from 4MMCA-PDA polyimine has better crystallinity with larger crystalline domain size.

TAPT-PDA COF

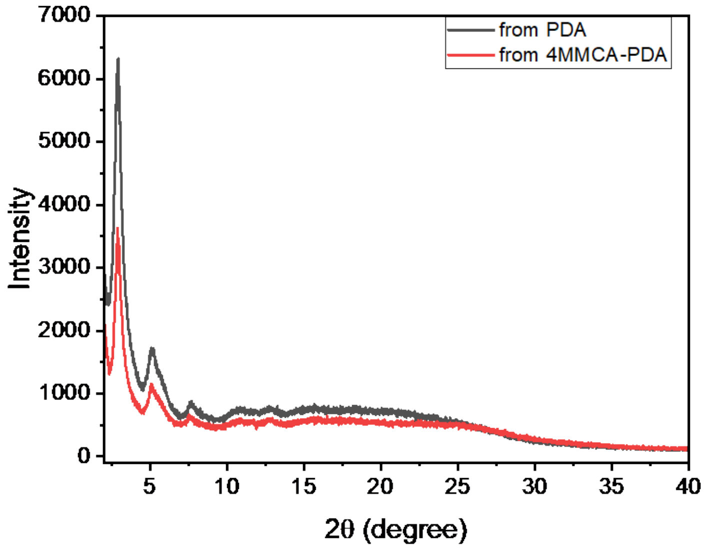

Figure S31. PXRDs of TAPT-PDA COF synthesized from PDA and from 4MMCA-PDA. FWHM analysis of the Bragg peak at 2.93° gave the value of 0.51 and 0.55 for COF synthesized from 4MMCA-PDA and PDA, respectively. The FWHM analysis indicated that the COF made from 4MMCA-PDA polyimine and PDA have similar crystallinity where the COF derived from 4MMCA-PDA has slightly larger crystalline domain size.

TAPB-F4PDA COF

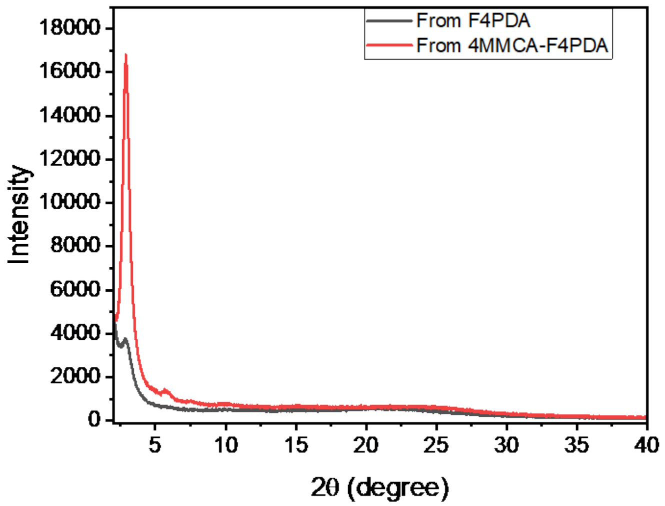

Figure S32. PXRDs of TAPB-F4PDA COF synthesized from F4PDA and from 4MMCA-F4PDA. FWHM analysis of the Bragg peak at 2.92° gave the value of 0.57 and 0.64 for COF synthesized from 4MMCA-F4PDA and F4PDA, respectively. Both intensity and FWHM analysis indicated that the COF made from 4MMCA-PDA polyimine have better crystallinity while F4PDA synthesis gave COF with low crystallinity. During the COF synthesis reaction of F4PDA and TAPB, insoluble polymer was formed before the addition of acetic acid catalyst, thus, hindering the distribution of acetic acid in the reaction media to catalyze COF forming reaction, resulting in low crystalline COF.

Gas Sorption

a)

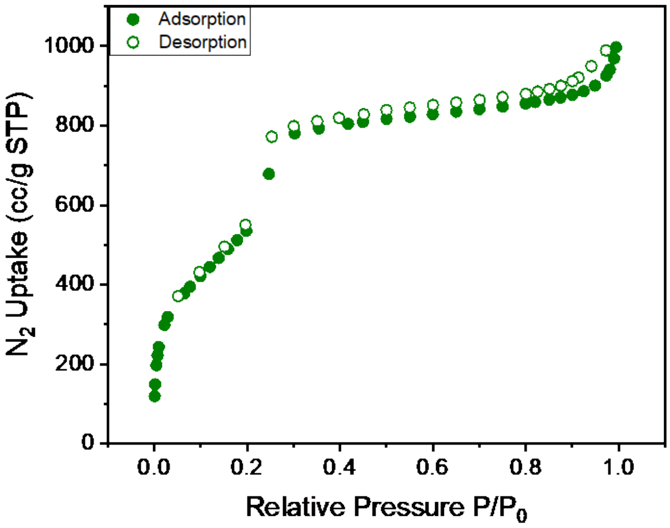

b)

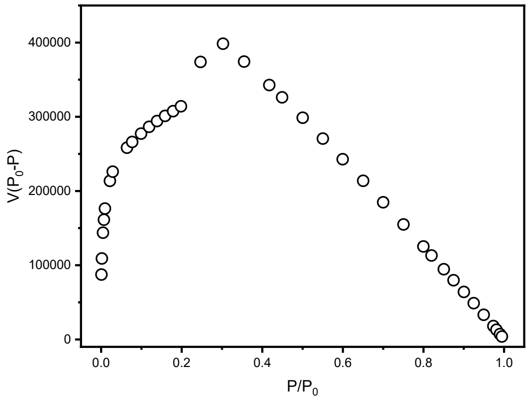

c)

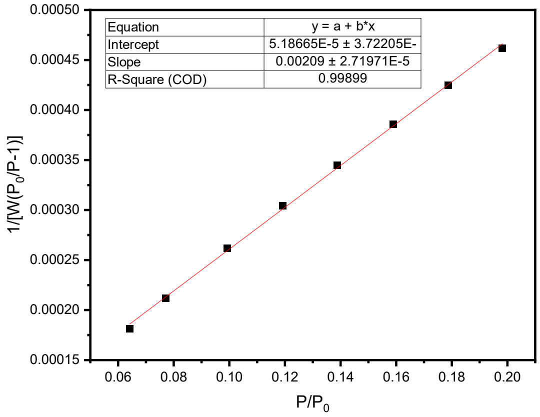

Figure S33. a) N_2 isotherm of TAPB-PDA COF made from 4MMCA-PDA and TAPB. b) Consistency criterion plot for determining P/P_0 range for BET analysis. [5,6] b) BET plot using points below $P/P_0 = 0.3$. The BET surface area was determined to be 2032 m^2 g^{-1}.

a)

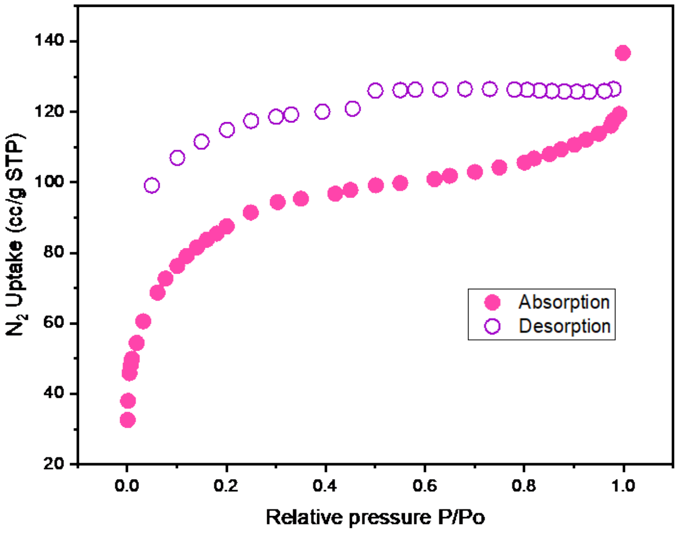

b)

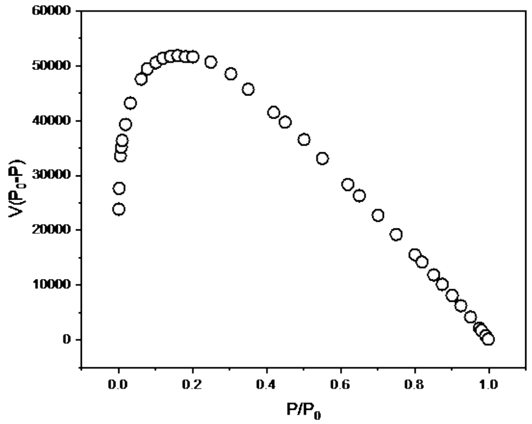

c)

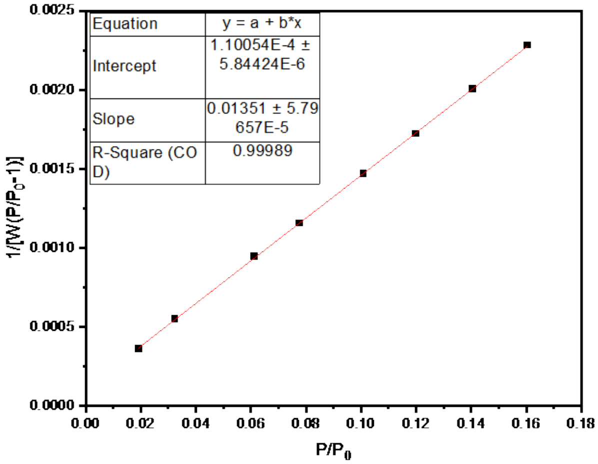

Figure S34. a) N_2 isotherm of TAPT-PDA COF made from 4MMCA-PDA and TAPT. b) Consistency criterion plot for determining P/P_0 range for BET analysis. [5,6] b) BET plot using points below $P/P_0 = 0.16$. The BET surface area was determined to be 320 m^2 g^{-1}.

a)

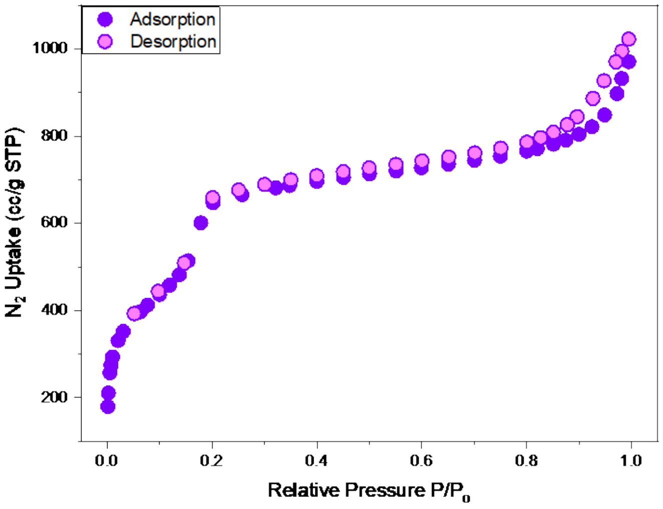

b)

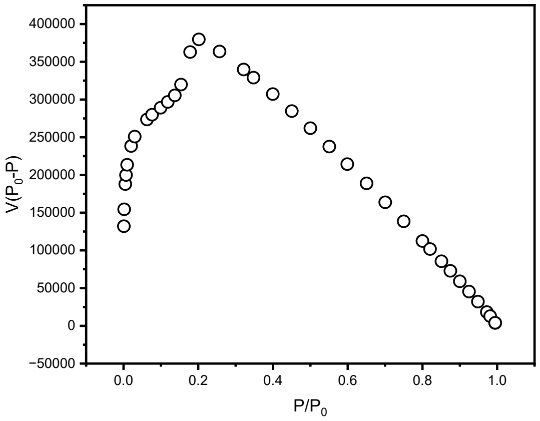

c)

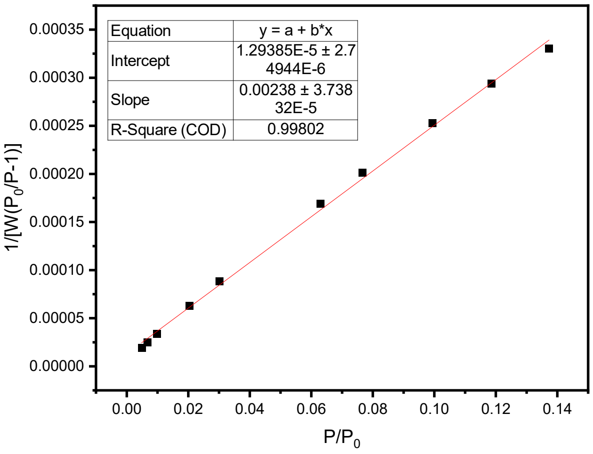

Figure S35. a) N_2 isotherm of ETTA-PDA COF made from 4MMCA-PDA and ETTA. b) Consistency criterion plot for determining P/P_0 range for BET analysis. [5,6] b) BET plot using points below $P/P_0 = 0.20$. The BET surface area was determined to be 1822 $m^2\ g^{-1}$.

a)

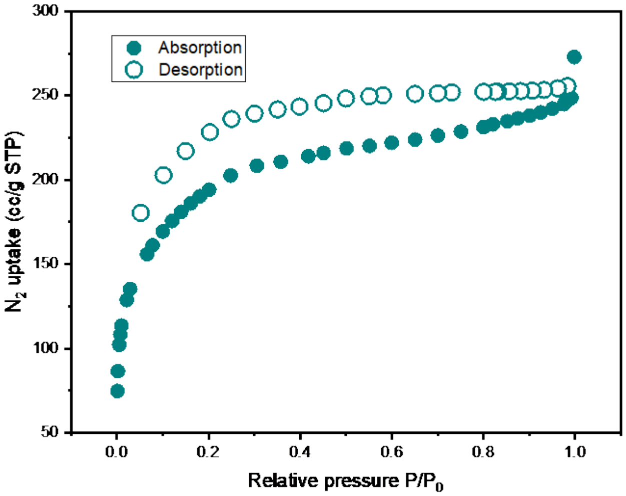

b)

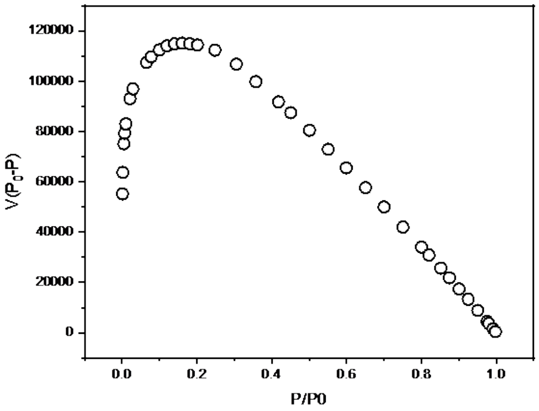

c)

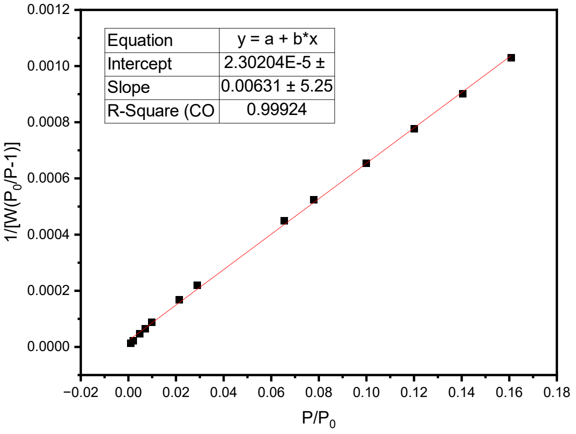

Figure S36. a) N_2 isotherm of TAPB-F4PDA COF made from 4MMCA-F4PDA and TAPB. b) Consistency criterion plot for determining P/P_0 range for BET analysis. [5,6] b) BET plot using points below $P/P_0 = 0.16$. The BET surface area was determined to be 688 $m^2\ g^{-1}$.

Porosity analysis of TAPB-PDA COF film synthesized from 4MMCA-PDA

Film 1: TAPB-PDA COF film was prepared following the description in COF in designed shape section in the Material synthesis part. Film 1 is a thin film.

Film 2: TAPB-PDA COF film was prepared as described in "**Stand alone thick COF film**" session in page S8.

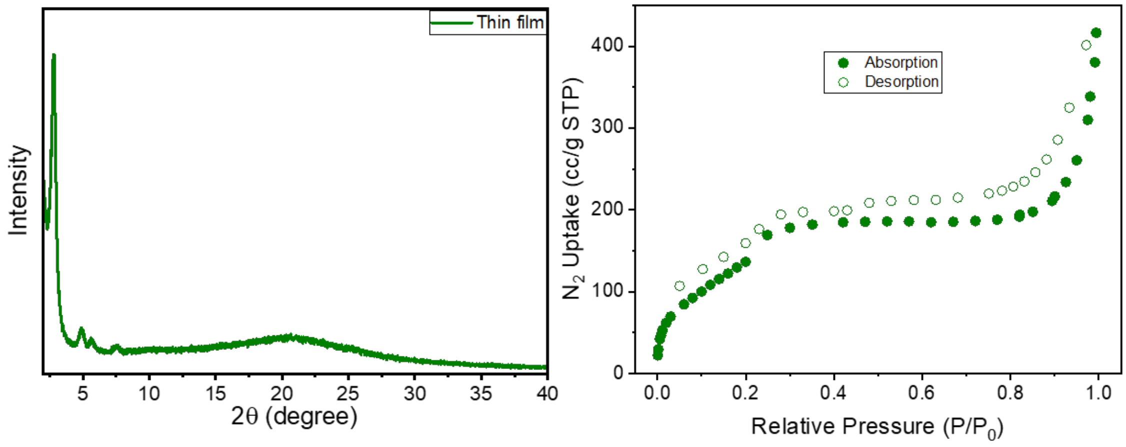

Figure S37. PXRD analysis of film 1 indicating crystallinity and N_2 sorption experiment of film 1. Calculation from the N_2 isortherm gave BET of 548 m^2/g. BET plot used 8 points in the range of $0.05 < P/P_0 < 0.2$.

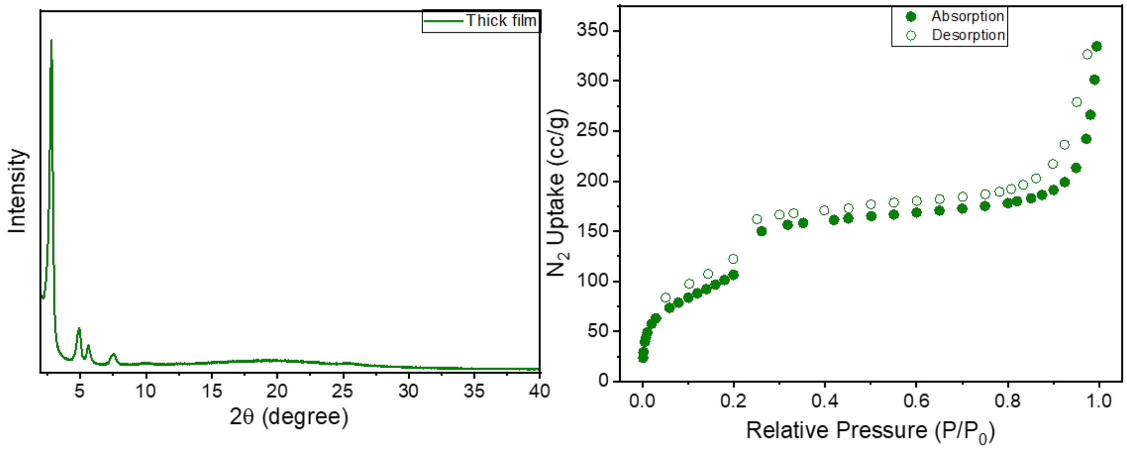

Figure S38. PXRD analysis of film 2 indicating crystalline COF and N_2 sorption experiment of film 2. Calculation from the N_2 isortherm gave BET of 401 m^2/g. BET plot used 8 points in the range of $0.05 < P/P_0 < 0.2$.

Raman Spectroscopy

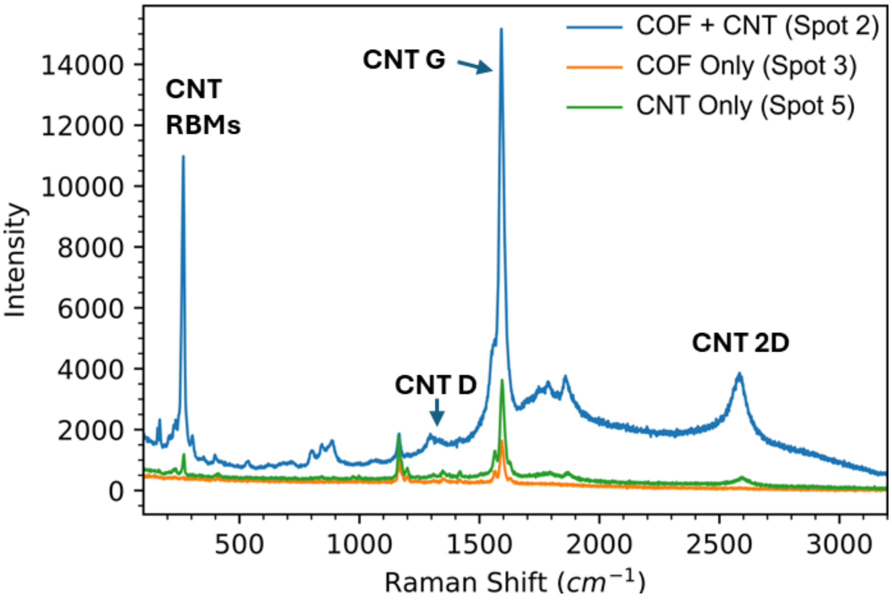

Figure S39. Raman spectra of COF/CNT-0.9 film where there are COF area, CNT area and COF + CNT area

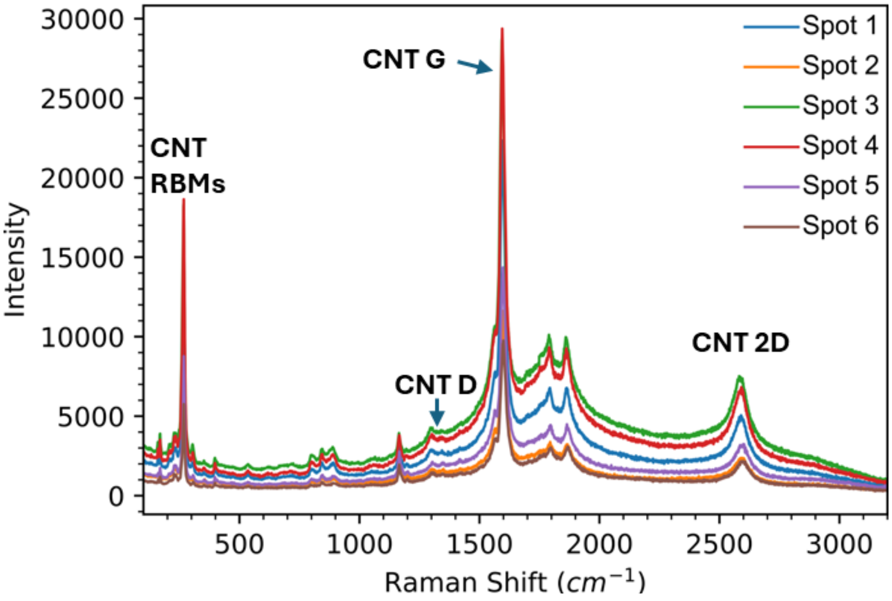

Figure S40. Raman spectra of COF/CNT-4.5 film where CNT can be found in all area of the film.

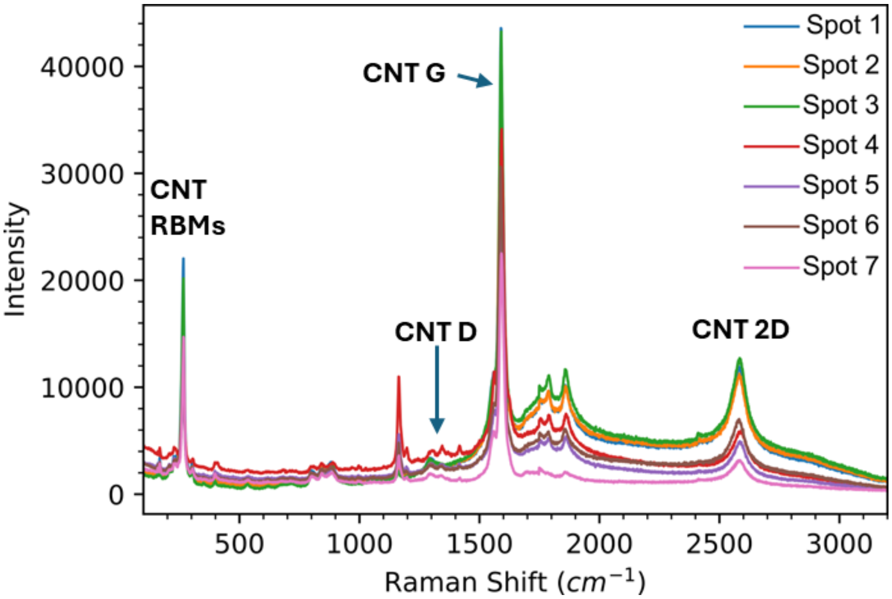

Figure S41. Raman spectra of COF/CNT-8.7 film where CNT can be found in all area of the film.

Resistivity Measurement

Table S4. COF conductivity for reported non-conductive systems and their composites with CNT and conductive polymers

Materials	Conductivity (S cm^{-1})	Resistivity (Ω· cm)	CNT loading	Notes	Ref
DAAQ-COF	3.23 E−9	3.10E+08	0		7
DAAQ-COF@CNT	1.48 E−6	6.76E+05	35 wt%	CNT-COOH	7
PPyrr-TPB-DMTP-COF	1.50E-05	6.67E+04	0	Polymerized Pyrrole	8
Pristine non-conductive COF	1.00E-13	1.00E+13			8
TTF-COF	1.2 E-4	8.33E+03			9
COF/CNT-0.9		84816.6	0.9 wt% in precursor film		This work
COF/CNT-4.5		15.21	4.5 wt% in precursor film		This work

Conductivity measurement was performed on the 4-point linear probe including the Signatone Probe connected to a Keithley 2400 Source Meter[6]

$$R_s = sC\frac{V}{I}\ (\Omega)$$

Where s is the distance between the probes 1 mm

C is a correction factor depends on the dimensions of the samples

$\frac{V}{I}$ can be calculated from the I(V) curve

Resistivity can be calculated by:

$$\rho = R_s w\ (\Omega{\cdot}cm)$$

Where w is thickness of the films (cm)

Table S5. Resistivity values of 4MMCA-PDA/TAPB/CNT and TAPB-PDA-COF/CNT films with different CNT loadings

Sample	w (μm)	C	ρ (Ω·cm)
4MMCA-PDA-TAPB-CNT-8.7%	5.308	4.48	2.045
4MMCA-PDA-TAPB-CNT-4.5%	8.152	4.49	97.17
4MMCA-PDA-TAPB-CNT-0.9%	3.04	4.49	22686.06
COF/CNT-8.7	8.75	4.24	3.25
COF/CNT-4.5	3.98	4.22	15.21
COF/CNT-0.9	14.52	3.21	84816.6

Film thickness of 4MMCA-PDA/TAPB/CNT films were measured by contact profilometer.

Film thickness of TAPB-PDA-COF/CNT films were measured by SEM cross-sectional analysis

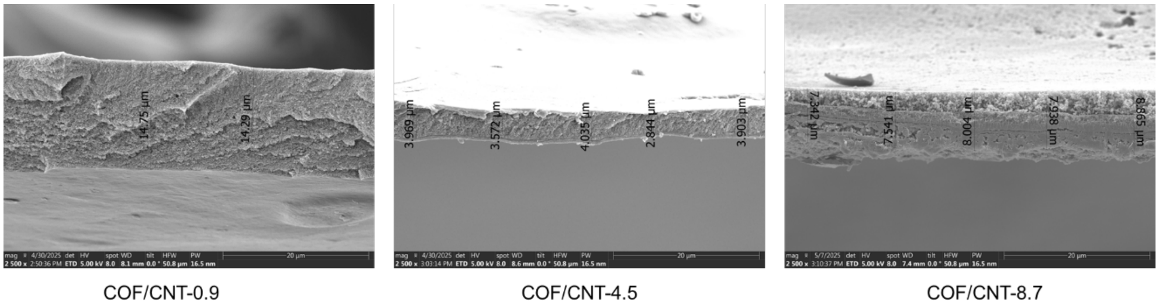

Figure S42. SEM images of cross-sectional of COF/CNT-0.9, 4.5, and 8.7. These measurements were used to estimate the thickness of the films.

Scanning Electron Microscopy

SEM images of thin films discussed in COF film synthesis and Figure 5 of the manuscript, before and after conversion to COF.

Precursor film composed of 4MMCA-PDA and TAPB mixture on glass slide

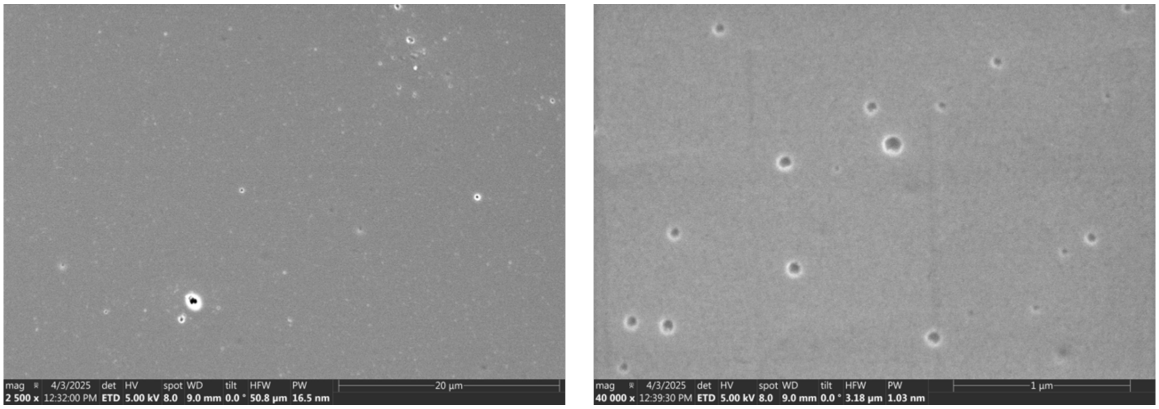

Figure S43. SEM images at 2500x and 40000x magnifications of film composed of 4MMCA-PDA and TAPB

Thin film of TAPB-PDA COF made from conversion of the precursor film composed of 4MMCA-PDA and TAPB

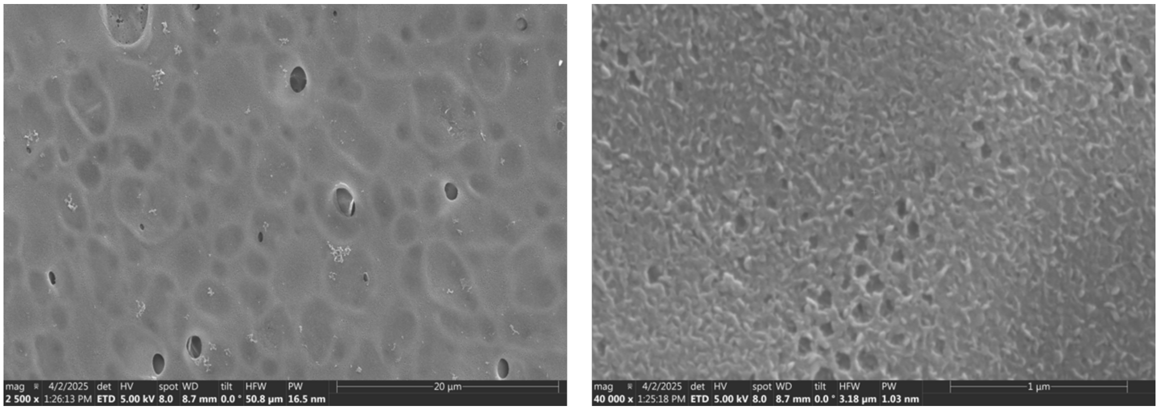

Figure S44. SEM images at 2500x and 40000x magnifications of TAPB-PDA COF film on glass substrate

SEM images of stand-alone films discussed in the first part of Functional manufacturing and Figure 6 of the manuscript, before and after conversion to COF.

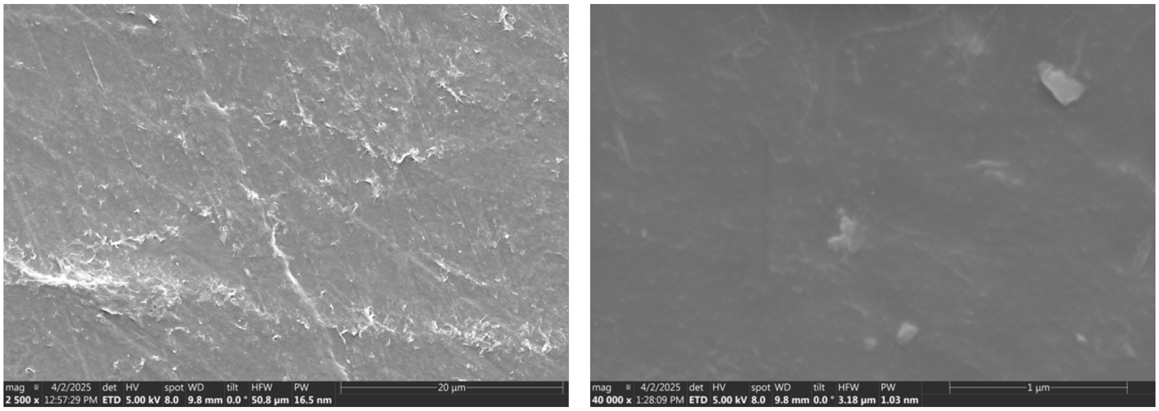

Figure S45. SEM images at 2500x and 40000x of the stand-alone composed of 2 layers: 4MMCA-PDA and 4MMCA-PDA+TAPB

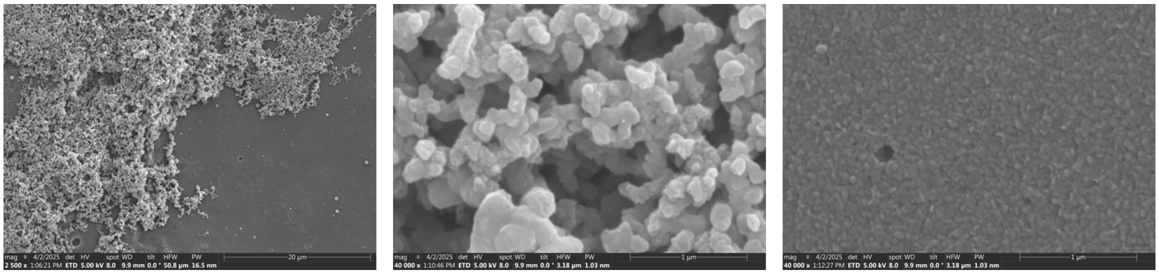

Figure S46. SEM images at 2500x and 40000x of the TAPB-PDA COF film synthesized from the stand-alone precursor film (left) film with rough and smooth regions, (middle) higher magnification of the rough region, (right) higher magnification of the smooth region.

Thickness measurement through SEM of thick film

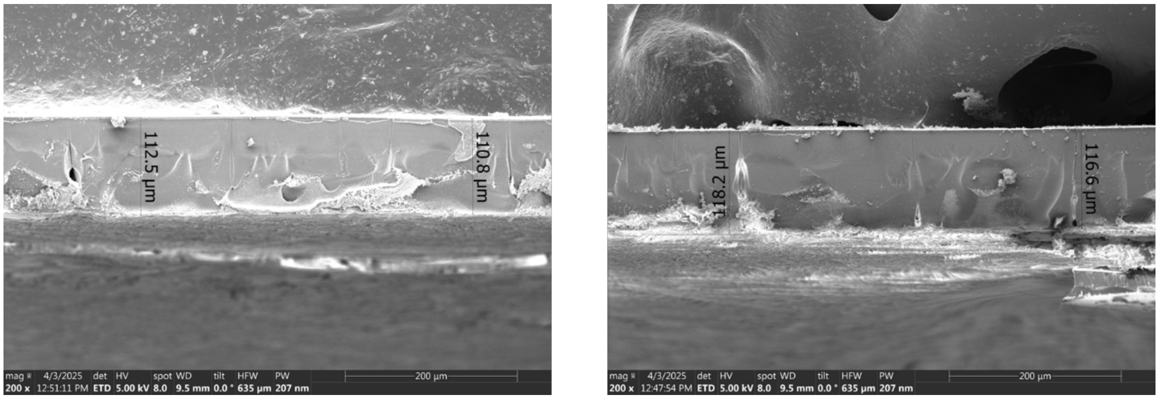

Figure S47. Cross-sectional SEM of thick TAPB-PDA COF film synthesized in this work. The film has thickness of 114.5 ± 3.0 µm

SEM Cross-sectional of TAPB-PDA-COF/CNT composite

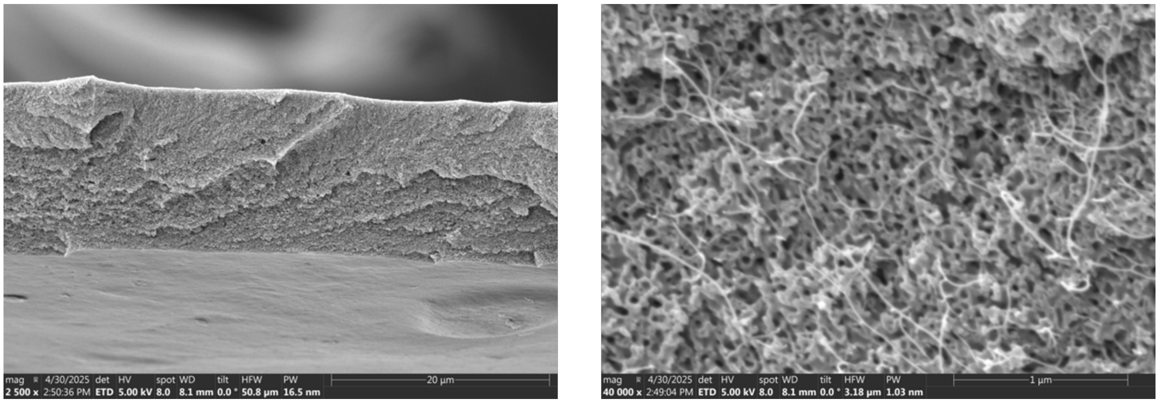

Figure S48. Cross-sectional SEM images at 2500x and 40000x magnification of COF/CNT-0.9 composite

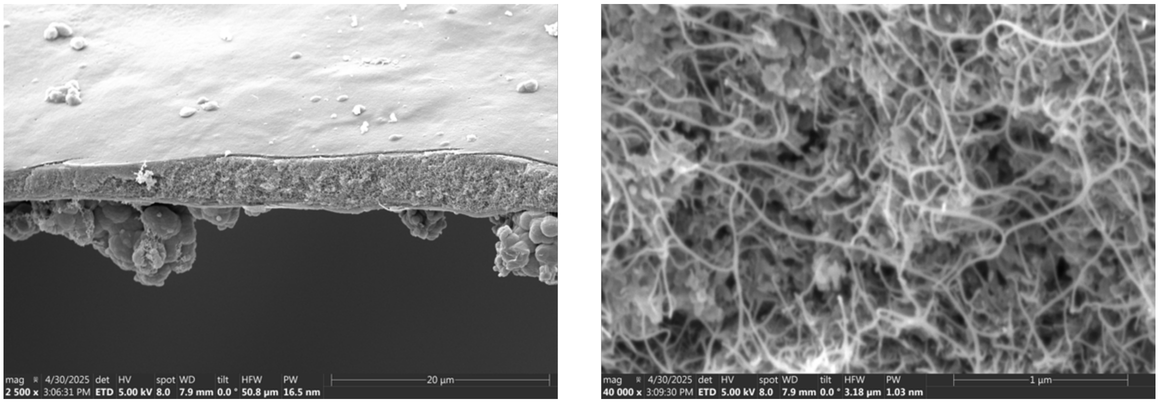

Figure S49. Cross-sectional SEM images at 2500x and 40000x magnification of COF/CNT-4.5 composite

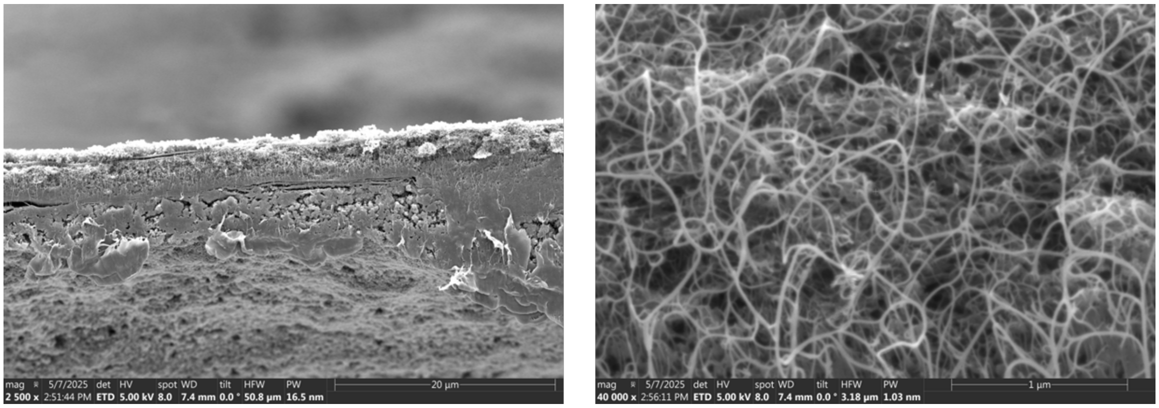

Figure S50. Cross-sectional SEM images at 2500x and 40000x magnification of COF/CNT-8.7 composite. The SEM image indicated inhomogeneity in the film.

References

1. Smieska, L. *et al.* The Functional Materials Beamline at CHESS. *Synchrotron Radiat. News* **36**, 4–11 (2023).

2. Stoupin, S. *et al.* Side-bounce beamlines using single-reflection diamond monochromators at Cornell High Energy Synchrotron Source. *J. Synchrotron Radiat.* **28**, 429–438 (2021).

3. Meaurio, E. Generalization of the Carothers equation for linear step growth polymers. *Sci. Rep.* **14**, 27281 (2024).

4. Cullity, B. D. & S. R. Stock. *Elements of X-Ray Diffraction*. (Prentice Hall, NJ).

5. Rouquerol, J., Llewellyn, P. & Rouquerol, F. Is the BET equation applicable to microporous adsorbents? in 49–56 (2021). doi:10.1016/s0167-2991(07)80008-5.

6. Walton, K. S. & Snurr, R. Q. Applicability of the BET Method for Determining Surface Areas of Microporous Metal−Organic Frameworks. *J. Am. Chem. Soc.* **129**, 8552–8556 (2007).

7. Duan, J. *et al.* Construction of a Few-Layered COF@CNT Composite as an Ultrahigh Rate Cathode for Low-Cost K-Ion Batteries. *ACS Appl. Mater. Interfaces* **14**, 31234–31244 (2022).

8. Gong, Y. *et al.* Wiring Covalent Organic Frameworks with Conducting Polymers. *Angew. Chemie Int. Ed.* **63**, e202411806 (2024).

9. Cai, S.-L. *et al.* Tunable electrical conductivity in oriented thin films of tetrathiafulvalene-based covalent organic framework. *Chem. Sci.* **5**, 4693–4700 (2014).
